# Stabilized Reconstruction of Signaling Networks from Single-Cell Cue-Response Data

**DOI:** 10.1038/s41598-019-56444-5

**Published:** 2020-01-27

**Authors:** Sunil Kumar, Xiao-Kang Lun, Bernd Bodenmiller, María Rodríguez Martínez, Heinz Koeppl

**Affiliations:** 1 Institute of Biochemistry, ETH Zurich, Zurich, 8093 Switzerland; 2 Institute of Molecular Life Sciences, University of Zurich, Zurich, 8057 Switzerland; 3 IBM Research Zurich, Zurich, 8803 Switzerland; 4 Department of Electrical Engineering and Information Technology, Technische Universität Darmstadt, Darmstadt, Germany; 5 Present Address: Sleepiz AG, Zurich, Switzerland; 6 Present Address: Wyss Institute for Biologically Inspired Engineering, Harvard University, Boston, MA 02115 USA

**Keywords:** Computational models, Network topology

## Abstract

Inferring cell-signaling networks from high-throughput data is a challenging problem in systems biology. Recent advances in cytometric technology enable us to measure the abundance of a large number of proteins at the single-cell level across time. Traditional network reconstruction approaches usually consider each time point separately, resulting thus in inferred networks that strongly vary across time. To account for the possibly time-invariant physical couplings within the signaling network, we extend the traditional graphical lasso with an additional regularizer that penalizes network variations over time. ROC evaluation of the method on in silico data showed higher reconstruction accuracy than standard graphical lasso. We also tested our approach on single-cell mass cytometry data of IFNγ-stimulated THP1 cells with 26 phospho-proteins simultaneously measured. Our approach recapitulated known signaling relationships, such as connection within the JAK/STAT pathway, and was further validated in characterizing perturbed signaling network with PI3K, MEK1/2 and AMPK inhibitors.

## Introduction

Cell signaling networks are responsible for correct processing and integration of exogenous cues. Deregulated signaling network often leads to deleterious outcomes, such as cancer or autoimmune diseases. With the advancement of experimental techniques to quantify protein abundances and post-translational modifications of proteins, it has become feasible to reconstruct the wiring diagram of biological networks by statistical means. In particular, mass cytometry allows us to monitor over 40 different proteins or protein modifications at single-cell resolution and hence provides unprecedented data for solving this inverse problem^1,2^. By using heavy-metal-labeled anti-bodies that yield narrow spectral profiles, mass cytometry does not suffer from spectral overlap unlike fluorescent-based methods^3,4^.

**Figure 1:**
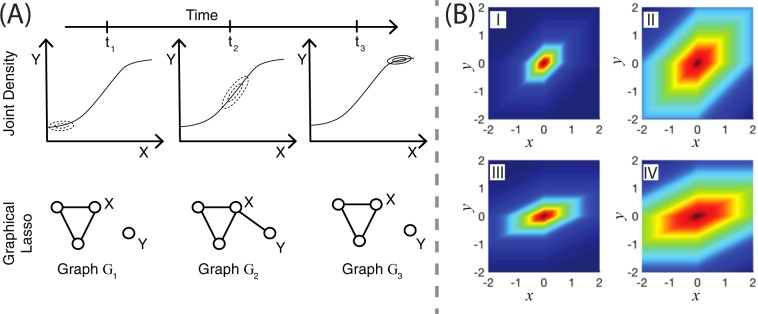
(**A**) Time-varying topology due to nonlinearities; (**B**) Prior interpretation and effect of data standardization on the prior distribution. (**A**) The joint density of the protein pair (x, y) at three different timestamps t1, t2 and t3 caused by a nonlinear response characteristics (upper row). The pair appears uncorrelated at times t1 and t3, but correlated at time t2. Traditional graphical lasso estimates conditional independence relations between them at each time point separately (lower row). Although, the relationship strengths between the protein pair (x, y) at timestamps t1 and t3 are low, the traditional graphical lasso model imposes high penalty to eliminate them. (**B**) The horizontal and the vertical axes represent variables x and y, respectively. The value of the function f(x,y) = exp(−α|βx − y| − α|βx| − α|y|) is indicated in the colored scale (low - blue; high - red). The quantities α and β denote the regularization and the scaling parameters, respectively. In (**I**) and (**II**), we keep the variables x and y in the same scale, i.e, β = 1, and show the impact of different levels of regularization. In (**III**) and (**IV**), we choose different scalings for x and y by setting β = 0.5. In figures (**I**) and (**III**), we set α = 1 (high regularization). In figures (**II**) and (**IV**), we set α = 0.25 (low regularization). The regularization strength impacts equally both variables if the scale parameter β is 1, as seen in figures (**I**) and (**II**). However, figures (**II**) and (**IV**) show that the impact of the regularization parameter on the variable x is diminished when it is scaled down by lowering β from 1 to 0.5.

**Figure 2:**
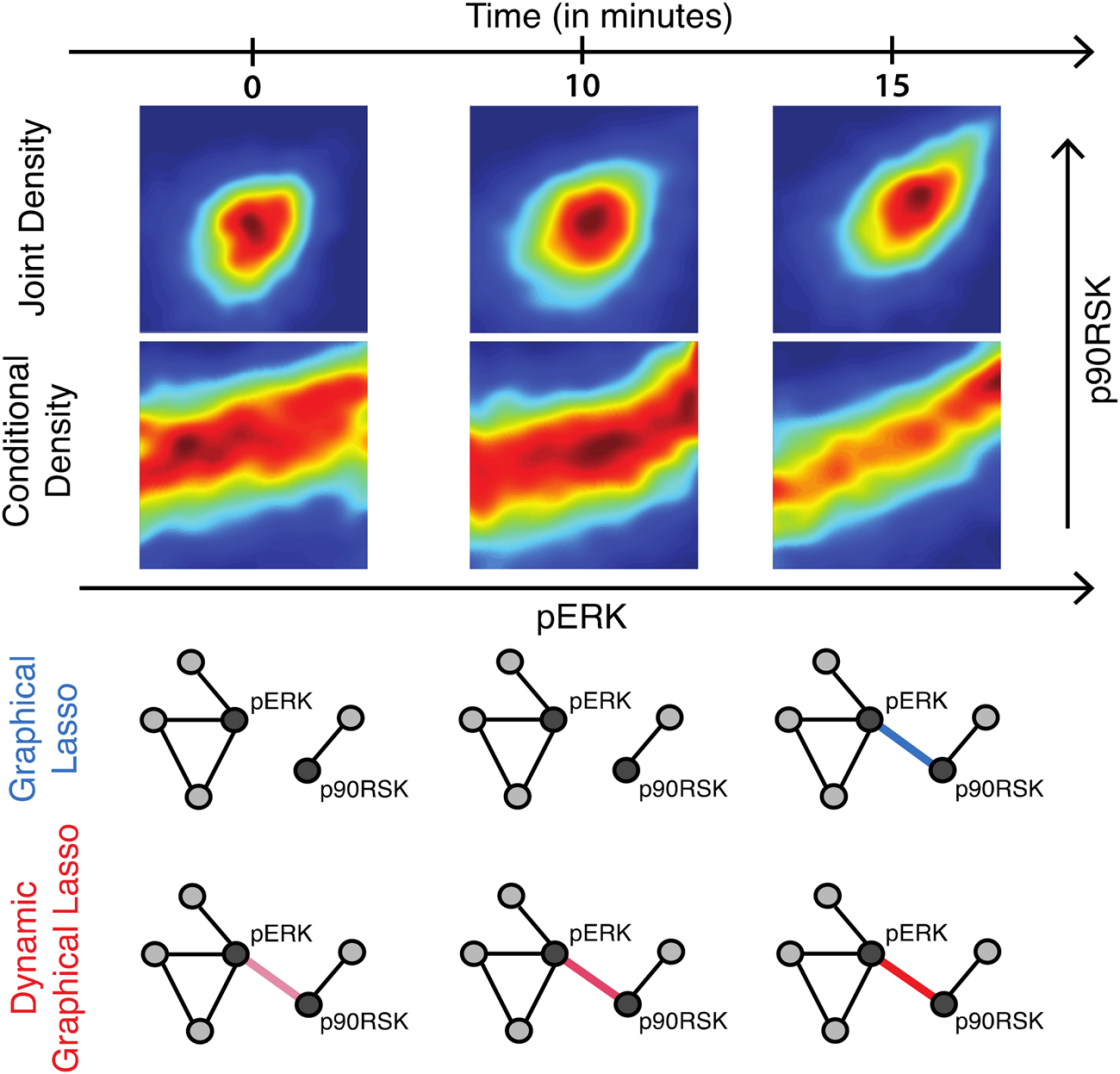
Experimental response characteristics. Phospho-ERK and Phospho-90RSK are measured at single-cell resolution using mass cytometry after IFN-γ stimulation. The first row shows the joint density plot between pERK and p90RSK at three different time points. The second row shows the conditional density estimate of Phospho-90RSK level after conditioning on the level of Phospho-ERK using Gaussian kernel density estimator. The conditional density plots clearly show a nonlinear relationship between Phospho-ERK and Phospho-90RSK. Topologies from graphical lasso show high variability over time whereas our proposed dynamic graphical lasso provides more stable topologies.

Nevertheless, the available panel of antibodies usually only covers a fraction of the proteins involved in the signaling network under study, and hence unresolved components may introduce confounding effects. For instance, an unresolved kinase that targets two measured proteins of the network can lead to confounding effect through spurious edge. Although an erroneously inferred interaction between the two proteins cannot be prevented unless the unresolved kinase is measured, such a confounder may leave a fingerprint in the data. In particular, a temporal variation of the kinase activity leads to a variation in the inferred interaction strength. Furthermore, the biochemically mediated interactions among proteins often exhibit a nonlinear cue-response relationship that also leads to an apparent time-varying interaction strengths (Figs. 1A and 2). Taken together, the presence of these effects could lead to the conclusion that the topology of the network is changing during the course of induction, even though the true underlying network is actually time-invariant. On top of that, cell-to-cell variability may introduce spurious edges in the network owing to the heterogeneity across the cell population.

Under a Gaussian model assumption, network reconstruction can be done by performing inference on the precision matrix^5^. The Graphical least absolute shrinkage and selection operator (GLasso) allows us to impose sparsity on the inferred graph through an L_1_ penalization^6,7^. Note that treating different time points separately fails to account for the often time-invariant nature of the physical coupling underlying the signal transduction. Moreover, the signal strength is often misinterpreted as a change in network topology (Fig. 2).

To obtain a stabilized reconstruction of the signaling network, in this paper, we introduce an additional regularization parameter into the GLasso framework to penalize drastic changes in the inferred networks over consecutive time points. We address the problem of model selection (choosing the values of the regularization parameters) by deriving the Bayesian Information Criterion (BIC) corresponding to this setting. We apply our generalized model, called Dynamic Graphical Lasso (DynGLasso), to reconstruct the signaling topology related to dedicated a IFNγ stimulation experiment from time-course mass cytometric data.

Note that Hara and Washio^8^, Danaher *et al*.^9^ and Wu *et al*.^10^ introduced methods to learn common substructure of multiple graphical Gaussian models. They, however, did not consider the temporal aspect of the graphs. In contrast, many authors such as Gibberd and Nelson^11^, Hallac *et al*.^12^ and Cai *et al*.^13^ considered the temporal aspect in the recent years, but they did not address the problem of model-selection. Monti *et al*.^14^, Gibberd *et al*.^15^ and Cai *et al*.^13^ have implemented the model on continuous time series data targeting the estimation of smooth inverse covariance matrices. In our case, we have designed a method that is applicable to datasets with limited timestamps such as mass cytometry to provide a qualitative understanding of the relationships between variables.

## Results

### Statistical methodology

We adopt a probabilistic graphical model in which the conditional dependence relationships among a set of *p* random variables are represented by edges of a graph over the *p* nodes. Consider the graphs ${{\mathscr{G}}}_{t}=(V,\,{E}_{t})$ over $V=\{1,2,\ldots ,p\}$, where *E*_*t*_ is the set of edges at time *t*. The zero elements of the corresponding adjacency matrix *A*_*t*_ imply conditional independence between two random variables. Under a Gaussian distributional assumption, the *i*-th and the *j*-th random variables are independent, conditional on the rest if the (*i, j*)-th element of the precision matrix is zero^5^.

To prevent large changes in the inferred graphs over consecutive time points, we introduce an additional regularization parameter into the traditional GLasso framework. Suppose we are given *n*_*t*_ independent samples at time *t* drawn from a multivariate Gaussian distribution ${\mathscr{N}}(0,{\Sigma }_{t}),\,t=1,2,\ldots ,T$. Let *S*_*t*_ be the standardized sample covariance matrix at time *t*. Also denote the tuple of precision matrices $({\Theta }_{1},{\Theta }_{2},\ldots ,{\Theta }_{T})$ by **Θ**. We define our objective function as

$$\Phi ({\boldsymbol{\Theta }})=\mathop{\sum }\limits_{t=1}^{T}\frac{{n}_{t}}{2}[\log ({\rm{\det }}({\Theta }_{t}))-{\rm{Tr}}({S}_{t}{\Theta }_{t})]-\frac{\lambda }{2}\mathop{\sum }\limits_{t=1}^{T}{\Vert {\Theta }_{t}\Vert }_{1}-\frac{\rho }{2}\mathop{\sum }\limits_{t=2}^{T}{\Vert {\Theta }_{t}-{\Theta }_{t-1}\Vert }_{1},$$

where λ ≥ 0, ρ ≥ 0, and perform the following optimization

1 $$\mathop{{\rm{\max }}}\limits_{{\boldsymbol{\Theta }}}\Phi ({\boldsymbol{\Theta }}),\,{\rm{subject}}\,{\rm{to}}\,{\Theta }_{t}\,\,\succ \,0\,{\rm{for}}\,t=1,2,\ldots ,T,$$

where ${\Theta }_{t}\,\succ \,0$ denotes positive definiteness of ${\Theta }_{t}$, and det and Tr, respectively, denote the determinant and the trace of a matrix. The sample covariance matrices are standardized to zero mean and unit variance to avoid the adverse effect of the variances on the penalization rates (see Fig. 1B). Mathematically, $\Phi (\,\cdot \,)$ is the log-likelihood function with two separate L_1_ penalizations. It is convex in **Θ**, and hence the optimization problem in Eq. (1) yields a unique optimal solution $\hat{{\boldsymbol{\Theta }}}=({\hat{\Theta }}_{1},{\hat{\Theta }}_{2},\ldots ,{\hat{\Theta }}_{T})$, where ${\hat{\Theta }}_{t}$ represents the estimated precision matrix at time *t*. The regularization terms λ and ρ are called the sparsity and the smoothing parameter, respectively.

**Figure 3:**
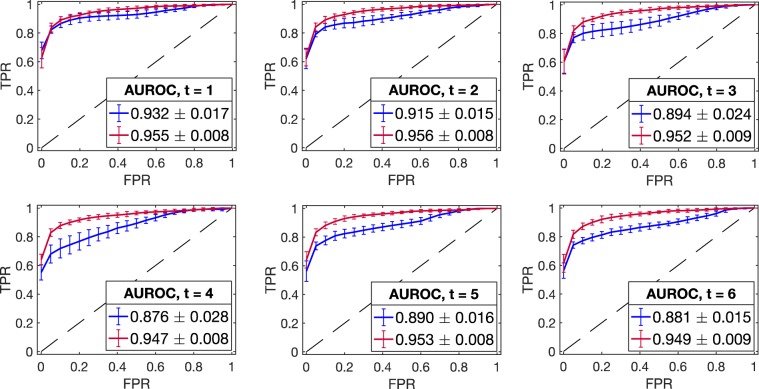
Model performance evaluation using ROC curves (*in silico* study). The mean ROC curve corresponding to DynGLasso (red line) is closer to the upper left corner than the model GLasso (blue line), indicating a higher accuracy in the network estimation. The mean ROC curves are estimated using 20 synthetic time-series datasets, and the error bars correspond to their standard deviation. The estimated AUROC (in the legend) shows that the performance of DynGLasso is higher than GLasso and the low standard deviation of AUROC indicates a higher robustness of DynGLasso predictions.

For λ = 0 and ρ = 0, the optimization problem in (1) yields the maximum likelihood (ML) estimate, provided $p\le {n}_{t}$ for all *t*. In the high-dimensional setting $(p > {n}_{t})$, the ML estimate does not exist as *S*_*t*_ is singular. A nonzero λ fixes this issue and imposes sparsity in the model^7^. We show later that $\lambda \ge {\lambda }^{\ast }$ (Eq. (2)) leads to totally disconnected graphs.

The smoothing parameter ρ is new and penalizes large changes over consecutive time points. We later show that for $\rho \ge {\rho }^{\ast }$ (Eq. (3)), the structural variation between the graphs becomes zero, leading to the same estimate for all time points. The structural variation decreases as the smoothing parameter increases (see SI.1 for details).

### Bayesian interpretation

The first sum appearing in Eq. (1) is the log-likelihood, and the terms involving the regularization parameters λ, ρ incorporate the prior information (namely, sparsity and smooth variation of the network over time). We take the product of two Laplace distributions as our prior. The first Laplace distribution leads to sparsity, as done in GLasso. The second Laplace distribution on the differences ${\Theta }_{t}-{\Theta }_{t-1}$ imposes smoothness in structural variation (see Fig. 1B). The optimal value $\hat{{\boldsymbol{\Theta }}}$ is the *maximum a posteriori* (MAP) estimate (see SI.2).

It is important to set the sparsity parameter λ suitably to avoid severe penalization. We follow a two-stage procedure mentioned in^16^. In the first stage, we connect two variables at time *t*, i.e., place an edge between them, if the corresponding entry of the estimated precision matrix is larger than a certain nonzero threshold. In the second stage, we re-estimate the precision matrices without penalizing the set of connected pairs selected in the first stage. We keep the smoothing parameter unchanged in the second stage to ensure smoothness (see algorithm 1 in SI.5).

### Model selection

The efficacy of our method depends on the choice of λ and ρ. We adopt the BIC as a model-selection algorithm. Following the Bayesian interpretation introduced in the previous section, we derive the following BIC score function (details of the derivation can be found in section SI.3):

$$\begin{array}{c}BIC(\lambda ,\rho )=\mathop{\sum }\limits_{t=1}^{T}-\frac{{n}_{t}}{2}[{\log ({\rm{\det }}(\hat{\Theta }}_{t}))-{\rm{Tr}}({S}_{t}{\hat{\Theta }}_{t})]\,+\mathop{\sum }\limits_{t=1}^{T}[\frac{{k}_{t}}{2}\,\log \,\frac{{n}_{t}}{2\pi }]\\ \,+\,\frac{\lambda }{2}\mathop{\sum }\limits_{t=1}^{T}{\Vert {\hat{\Theta }}_{t}\Vert }_{1}+\frac{\rho }{2}\mathop{\sum }\limits_{t=2}^{T}{\Vert {\hat{\Theta }}_{t}-{\hat{\Theta }}_{t-1}\Vert }_{1}-c(\lambda ,\rho ,T,p),\end{array}$$

where *k*_*t*_ is the number of unique nonzero elements in ${\hat{\Theta }}_{t}$. The term $c(\lambda ,\rho ,T,p)$ is an approximate model-specific, data-independent constant factor derived using the prior distribution. It is intended to remove bias induced by the estimates. We validate the BIC using the MCMC algorithm on synthetic data (see SI.7, SI.8). The iterative steps to calculate $c(\lambda ,\rho ,T,p)$ are explained in the Supplementary Information SI.4.

Numerical experiments demonstrate that there exist two critical values, ${\lambda }^{\ast }$ and ${\rho }^{\ast }$, above which only trivial outcomes (fully disconnected and static networks, respectively) are observed. We thus restrict the search space to $(0,{\lambda }^{\ast }]\times (0,{\rho }^{\ast }]$, where

2 $${\lambda }^{\ast }={{\rm{\max }}}_{t,i,j,i\ne j}{n}_{t}{S}_{t}(i,j),$$

3 $${\rho }^{\ast }={{\rm{\max }}}_{t}({n}_{t},\,{n}_{t-1}){{\rm{\max }}}_{t,i,j,i\ne j}({S}_{t}(i,j)-{S}_{t-1}(i,j)),$$

where ${S}_{t}(i,j)$ is the (*i, j*)-th element of *S*_*t*_. Within this restricted space, we follow a greedy approach to choose $(\lambda ,\rho )$ with the least $BIC(\lambda ,\rho )$ score.

Next, we compare our approach based on the BIC score with the commonly used cross validation (CV) approach for model selection. We carried out a 10-fold CV to choose $(\lambda ,\rho )$. We observed that, in agreement with previously published results (see^17^ and SI.9.2), BIC is more effective than CV in imposing sparsity. This is because BIC directly penalizes the number of nonzero elements of the precision matrices while CV aims to optimize the prediction accuracy without specifically trying to find sparse models. Moreover, CV is computationally more expensive, and hence not suitable for high dimensional problems^17^, and it has been reported to give inconsistent results for model selection^18^. Please note that the expression for the Kullback-Leibler divergence (D_KL_, also known as entropy loss) obtained for GLasso^17^ is not applicable in our setting, as it does not consider the temporal aspect of the datasets.

### *In silico* experiments

In our first experiment, we considered networks with 30 nodes and six time points. We randomly generated an undirected network ${{\mathscr{G}}}_{1}$ by imposing a targeted sparsity level. Afterwards, we assigned weights to each edge from a uniformly generated random variable over the range (−1, −δ] ∪ [δ, 1). This gives us a weighted directed adjacency matrix *W*. The threshold δ = 0.2 is chosen to avoid weak edges. The nonzero entries *W*_*ij*_ are interpreted as a directed edge from *X*_*j*_ to *X*_*i*_ with weight *W*_*ij*_. Finally, we obtained the precision matrix using the following transformation ${P}_{1}={(I-W)}^{T}(I-W)$, where *I* is the identity matrix. The transformed matrix *P*_1_ satisfies the properties of a precision matrix, i.e., it is symmetric and positive definite. The precision matrices of subsequent time points were obtained by adding randomly generated positive definite symmetric matrices whose elements are small in magnitude. For all precision matrices we kept the level of sparsity in the range 75% to 80%. We simulated 20 datasets from a Gaussian model, each containing *n*_*t*_ samples for each time point *t*. Then, we added Gaussian noise to recapitulate the noisy nature of biological experiments (see SI.9.1 for details). For this specific study, we considered *n*_*t*_ = 1000. We also explored the effect of sample size in the next subsection.

We applied our model DynGLasso to the generated datasets and used a ROC (Receiver Operating Characteristic) curve^8^ to compare the predicted graphs against the true ones. The ROC curve is a graphical plot that illustrates the performance of the model in terms of the TPR (True Positive Rate) and the FPR (False Positive Rate) as a discrimination threshold on the partial correlation is varied. We compared the DynGLasso against GLasso separately for each time point by plotting the mean ROC curve, averaged over 20 datasets, and measuring the area under the ROC curve (AUROC) (see Fig. 3). The error bars in the Fig. 3 indicate the standard deviation of the ROC curve. The AUROC for GLasso (blue line) is lower than the AUROC for DynGLasso (red line), indicating that DynGLasso achieves a higher performance than GLasso. Moreover, the smaller error bars for DynGLasso indicate that the smoothing parameter strengthens the consistency of the estimates.

**Figure 4:**
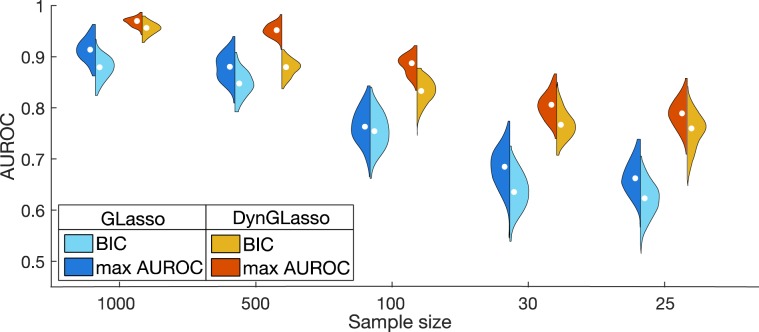
*In silico* study under the high-dimensional setting $({n}_{t} < p^{\prime} \,{\rm{where}}\,p^{\prime} =p(p+1)/2,$ total number of free parameters). According to the AUROC estimates, the proposed model DynGLasso performs better than GLasso under the high-dimensional setting. This plot shows the kernel density estimate of AUROCs from 20 distinct time-series datasets. When the sample size is large, the BIC estimates converge to the estimates with max AUROC. The max AUROC is the maximum AUROC computed over the hyperparameter space via grid search.

### Performance in the high-dimensional setting

To test the performance of DynGLasso in the high dimensional setting $(p^{\prime}  > {n}_{t})$, we varied the sample size from 1000 to 25 and estimated the precision matrices using the BIC score function for 20 different datasets. For given $p,\,p^{\prime}$ is equal to $p(p+1)/2$ which is same as the number of free parameters in the inverse covariance matrix. Even when the number of features is higher than the number of samples $(p^{\prime}  > {n}_{t})$, DynGLasso consistently outperformed GLasso (see Fig. 4). Moreover, GLasso performance showed a steeper decrease with same size than DynGLasso performance. Even though BIC is an approximation of the negative logarithm of the model posterior density, it led to estimates with high AUROC on average for large sample sizes, demonstrating that the BIC provides a reasonable approximation for large sample size. We also evaluated the impact of noise variance and observed that the AUROC estimate of DynGLasso was larger than that of GLasso for low noise variance, while for high noise variance they coincide (see SI.9.3 for details).

**Figure 5:**
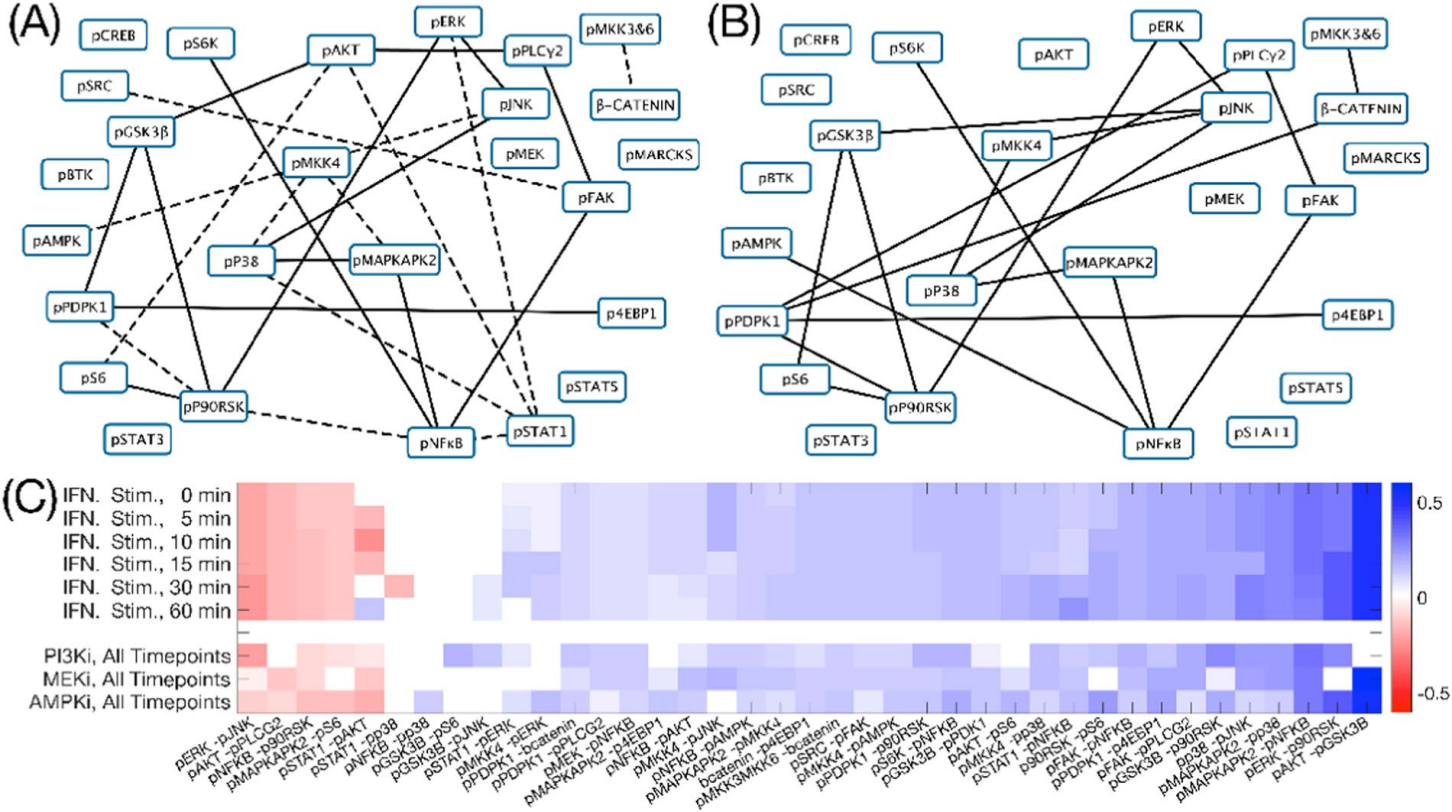
Network reconstruction from IFNγ stimulation experiments. **(A)** Reconstructed signaling pathway from the IFNγ stimulated time-course experiment. **(B)** Reconstructed signaling pathway from a time-course experiment that combined stimulation of IFNγ and PI3K inhibition (reconstructed signaling pathways from other inhibition time-course experiments are shown in the Supplement Fig. SI.11.7). For both figures **(A,B)**, the edges with solid lines represent stable undirected relationships between proteins across all time-points and the dashed lines connect protein pairs that are partially correlated only for some time points. **(C)** Heatmap of the estimated partial correlation using all four available mass-cytometry time-course datasets. All datasets are IFNγ stimulated and three additional experiments are perturbed with inhibitors of PI3K, AMPK and MEK1/2 for validation purposes. The heatmap shows the estimated partial correlation of the union of top 20 protein-pairs selected from different biological experiments.

### Application to mass cytometry data

To demonstrate a real-life application of DynGLasso, we reconstructed a signaling network from mass cytometry data. We recorded abundances of 26 phospho-proteins at single-cell resolution of the THP1 cell line. Cells were incubated with Interferon-γ (IFNγ) for time courses of 0, 5, 10, 15, 30 and 60 min (see the section “Materials and Methods” for experimental details and SI.11).

IFNγ plays a fundamental role in macrophage activation, T-helper cell response, inflammation and host defense against intracellular pathogens^19^. IFNγ signaling initiates from the ligand-receptor binding that activates intracellular signal transduction through the Janus kinase (JAK)/signal transducers and activators of the transcription (STAT) pathway. Subsequently, STAT1 translocates into the nucleus where the alteration of transcription occurs^20^. Moreover, IFNγ also induces gene expression via STAT1-independent pathways and modulates various signaling responses via the MAPK/ERK cascade, the MAPK/p38 cascade and the PI3K/AKT axis^21^.

The reconstructed network from IFNγ-stimulated THP1 cells and the strength of relationships in terms of partial correlation are shown in the sub-Fig. 5A,C respectively. In the following, we summarise some of our findings.

In agreement with a previous study^22^, the strength of relationship between p-ERK and p-STAT1 increased over the first 30 min after stimulation and decreased afterwards. Interestingly, the relationship strength from p-ERK to p-p90RSK, and further from p-p90RSK to p-S6 increased over the period of 1 h, suggesting a more sustained signaling through crosstalk to MAPK/ERK cascade, compared to the canonical IFNγ-induced JAK/STAT pathway.

The mitogen-activated protein kinase kinase 4 (MKK4) directly phosphorylates the c-Jun NH2-terminal kinase (JNK) and p38 kinase in response to cellular stresses and pro-inflammatory cytokines^23^. In our IFNγ stimulation experiment, the strength of the relationship between p38 and p-MKK4 increased over the period of 1 h, while the strength of relationship between p-MKK4 and p-JNK was initially high, but diminished over time, indicating the differential signaling regulatory mechanisms between these two MAPK cascades.

### Validation using inhibition experiments

To validate the signaling relationships captured by our method, we inhibited THP1 cells with three different kinase inhibitors (PI3K inhibitor: GDC-0941, MEK1/2 inhibitor: CI-1040 and AMPK inhibitor: Compound C) before IFNγ stimulation. The PI3K inhibitor impedes the phosphorylation of AKT, resulting in weaker relationships between p-AKT and many downstream targets, including p-GSK3β (Fig. 5B), as expected^24^.

Adenosine monophosphate-activated protein kinase (AMPK) has a critical role in regulating growth and reprogramming metabolism^25^. We used an AMPK inhibitor (compound C) to validate the intervention between the measured p-AMPK and the downstream targets of AMPK. For instance, with the inhibition, strength of signaling relationship between p-AMPK and p-JNK dropped, recapitulating the previously known signaling connection^26^.

MEK1/2 is a central signaling protein in the MAPK/ERK cascade as it interacts with the downstream kinase ERK to regulate cell proliferation and survival. Expectedly, the level of p-ERK decreased after inhibiting MEK1/2 using CI-1040. Further, relationships between p-ERK and the downstream targets, including p-p90RSK and p-GSK3β were largely reduced (Fig. 5C), suggesting the influence of MEK1/2 inhibition on both the canonical signaling cascade and the crosstalk signaling^27^.

### Literature based validation

We validated the interactions estimated via DynGLasso (see Supplement SI.11) with Omnipath, a comprehensive database of literature-curated human signaling pathways^28^. We used Omnipath to identify the shortest directed path of all reconstructed interactions in the IFNγ stimulation network. Most of the interactions (16 out of 27) are identified as direct relationships, and others have a shortest directed path of length up to two. The full list of interaction paths are provided in the Supplementary Table SI.11.1.

Some of the relationships, such as p-MKK4 to p-AMPK and p-FAK to p-PLCγ2, are indirectly connected to each other via unmeasured proteins. As discussed in the introduction, indirected connections, without the intermediate signaling proteins being experimentally measured, can be detected as strong relationships with DynGLasso.

## Discussion

Motivated by biological systems in which the couplings among the agents remain time-invariant, we generalized the traditional GLasso by introducing an additional regularization parameter to prevent large changes in the inferred graph over consecutive time points. We call the generalized model DynGLasso. We derived the corresponding BIC score function and used it for model-selection. To test the efficacy of our method, we conducted two experiments - one on synthetic data and one on single-cell mass-cytometry experimental data. Our experimental results show that DynGLasso consistently outperforms GLasso and is suitable in the high-dimensional setting. DynGLasso has also been able to retrieve nonlinear relationships among signaling transduction pathways. Moreover, it uncovers insightful dependence relationships among a set of 26 phospho-proteins at single-cell resolution.

Our objective function for DynGLasso in (1) involves an L_1_ penalization. The rationale is that L_1_-norm forces a majority of the entries of the estimated precision matrix to be exactly zero. We also evaluated the performance of our model after replacing the smoothing penalization using block sparsity. The block L_2_ penalization will shrink a majority of the entries, but fails to produce exact zeros (see SI.10 for details).

Mass cytometry facilitates high-dimensional, quantitative analysis of protein expression at single-cell resolution. All individual cells were vaporized, atomized and ionized by inductively coupled plasma before their expression levels were measured. As the cells were completely disintegrated during the analysis, the cytometer could not record the resolution of a fixed cell over time. We could bypass this problem as DynGLasso only requires aggregate information, namely, the sample covariance matrices, as an input.

The derivation of the revised BIC is one of our main contributions. We adopted a greedy search in the parameter space to minimize the BIC score. We hope to find a dedicated algorithm for the choice of regularization parameters in a future work. It will also be interesting to incorporate prior information about the structure of the network itself into our objective function to achieve a potentially improved algorithm.

## Materials and Methods

### Cell culture

The THP-1 cell line, obtained from ATCC, was cultured in the RPMI-1640 Medium (52400025, Gibco) supplemented with 10% FBS, 100 U/ml penicillin and 100 μg/ml streptomycin.

### Kinase inhibition and IFNγ stimulation

PI3K inhibitor (GDC-0941), MEK1/2 inhibitor (CI-1040) and AMPK inhibitor (Compound C) were dissolved in DMSO at a concentration of 10 mM. To perform inhibition experiments, THP-1 cells were split into four T25 flasks. Inhibitors were added to the flasks at the final concentration of 10 μM. A control flask of THP-1 cells was supplemented with DMSO at the same volume. Two hours after inhibitors had been applied, cells from each flask were further split into a 6-well plate with 1 ml cell suspension per well. Cells were incubated with IFNγ (final concentration 100 ng/ml) for a time-course of 0, 5, 10, 15, 30 and 60 min (stimulation was performed in reverse order to enable simultaneous harvesting of all conditions). 5-iodo-deoxycytidine (IdU) was added to the medium at the final concentration of 10 μM 20 min before harvesting. At the end of the time course, paraformaldehyde (PFA, Electron Microscopy Sciences) was added to the cell suspension at a final concentration of 1.6%, and the mixtures were incubated at room temperature for 10 minutes. Cross-linked cells were washed twice with cell-staining media (CSM, PBS with 0.5% BSA, 0.02% NaN3), and after removal of supernatant, ice-cold methanol was used to resuspend the cells, followed by a 10-min permeabilization on ice or for long-term storage at −80 °C.

### Antibody conjugation

To generate isotope-labeled antibodies, the MaxPAR antibody conjugation kit (Fluidigm) was used with the manufacturer’s standard protocol. Antibody yielding was determined based on absorbance of 280 nm. For long-term storage of antibodies at 4 °C, Candor PBS Antibody Stabilization solution (Candor Bioscience GmbH) was applied to dilute antibodies.

### Barcoding and staining protocol

Formalin-crosslinked and methanol-permeabilized cells were washed three times with CSM and once with PBS. Cells were incubated in PBS containing barcoding reagents (102 Pd, 104 Pd, 105 Pd, 106 Pd, 108 Pd, 110 Pd, 113In and 115In) at a final concentration of 50 nM for 30 min at room temperature and then washed three times with CSM^29^. Barcoded cells were then pooled and stained with the metal-conjugated antibody mix at room temperature for 1 h^30^. The antibody mix was removed by washing the cells three times with CSM and once with PBS. For DNA staining, iridium-containing intercalator (Fluidigm) was diluted in PBS with 1.6% PFA and incubated with the cells at 4 °C overnight. On the day before measurement, the intercalator solution was removed and cells were washed with CSM, PBS, and ddH_2_O. After the last washing step, the cells were resuspended in ddH_2_O and filtered through a 70-μm strainer.

### Mass-cytometry analysis

EQ^TM^ Four Element Calibration Beads (Fluidigm) were added to the cell suspension at a 1:10 ratio (v/v). Samples were analyzed on a Helios (Fluidigm). The manufacturer’s standard operation procedures were used for acquisition at a cell rate of ≈200 cells per second. After acquisition, all FCS files from the same barcoded sample were concatenated. Data were then normalized, and bead events were removed^31^ before doublet removal and de-barcoding of cells into their corresponding wells using a doublet-filtering scheme and single-cell deconvolution algorithm^32^. Subsequently, data was processed using Cytobank (http://www.cytobank.org/). Additional gating on the DNA channels (191Ir and 193Ir) was used to remove remained doublets, debris and contaminating particulate. Cell-cycle and cell-volume effects were corrected computationally using CellCycleTRACER^33^.

### Mathematical derivation and algorithms

The derivation of the BIC score function is available in SI.3. The algorithms for DynGLasso and the MCMC validation of the BIC score are provided in the SI appendices SI.5 and SI.8, respectively. Additional information about the synthetic data study are available in SI appendix SI.9.

## Supplementary information

Supplementary Information

## Data Availability

Additional data and materials are available online. The code is available at https://git.rwth-aachen.de/bcs/dynglasso.
