# Supplementary material for: Stabilized Reconstruction of Signaling Networks from Single-Cell Cue-Response Data
Source: Sci Rep. 2020 Jan 27;10:1233. doi: 10.1038/s41598-019-56444-5 (PMC6985232; doi:10.1038/s41598-019-56444-5)
Supplement: Supplementary file 1 — Supplementary Information [file 41598_2019_56444_MOESM1_ESM.pdf]

Stabilized Reconstruction of Signaling Networks from Single-Cell Cue-Response Data

S. Kumar, X. Lun, B. Bodenmiller, M. R. Martínez, H. Koeppl

November 24, 2019

SI.1 Smoothing parameter

The dynamic graphical lasso is an extended version of graphical lasso (GLasso) that maximizes the L_1-penalized log-likelihood of the time-series dataset in the model. We assume that the observation from a time-series dataset follows a multivariate Gaussian distribution. The non-zero entries of the corresponding precision matrix encode the conditional dependence relationship among variables. The smoothing parameter in the model is introduced to incorporate structural variation of the networks over time. This variation is computed as the sum of the element-wise absolute difference of the estimated precision matrices over two consecutive time points. In fig. S.1.1, we show the effect of the smoothing parameter on a prior distribution of the model. The prior distribution without a smoothing parameter leads to sparse network estimation over time. If the prior probability along positive diagonal axis is low, then it indicates that the estimated networks are highly variant over time.

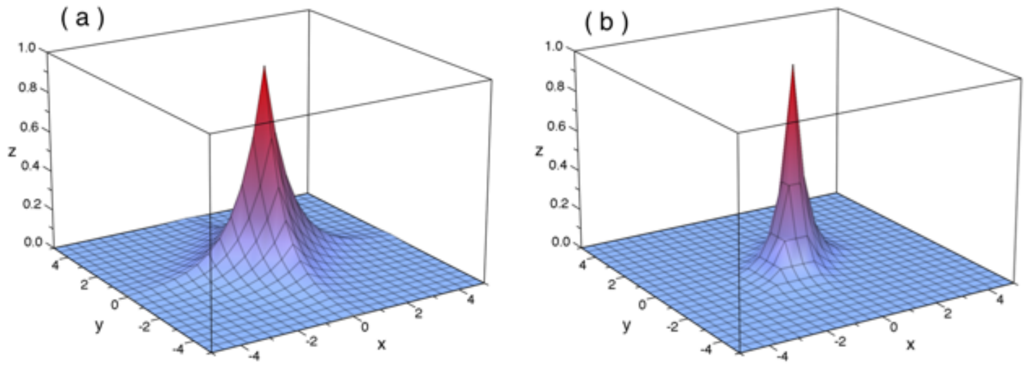

Figure S.1.1: Effect of smoothing parameter on the prior distribution. (a) Graph with sparsity parameter only, $f(x, y) = \exp(-|x| - |y|)$. **(b)** Graph with both parameters, sparsity and smoothing, $f(x, y) = \exp(-|x| - |y| - |x - y|)$. After adding a smoothing parameter to the model, the probability increases along the main diagonal axis, leading to smooth estimation over time.

SI.2 Mathematical problem formulation

Our objective function can also be given a Bayesian interpretation. By means of the Laplace approximation of marginal likelihood, the solution to the optimization problem is the *maximum a posteriori* (MAP) estimate of the posterior distribution of the time-series dataset.

The estimated undirected networks depend on the regularization parameters, sparsity and smoothing. The sparsity parameter enforces the networks to be sparse and the smoothing parameter minimizes the structural variation of the networks over consecutive time points. Let Θ_t denote the precision matrix at time point t. We denote the model parameters by $\boldsymbol{\Theta} \equiv (\Theta_1, ..., \Theta_T)$ and the model by $\mathcal{M} \equiv (\lambda, \rho)$. We take the prior distribution for the model as

$$P(\mathcal{M}, \boldsymbol{\Theta}) \propto \exp\left(-\frac{\lambda}{2}\sum_{t=1}^{T}||\Theta_t||_1 - \frac{\rho}{2}\sum_{t=2}^{T}||\Theta_t - \Theta_{t-1}||_1\right).$$

Let Y_t be the observation matrix at time point t of size $p \times n_t$, where n_t is the number of observations and p is the number of variables. If the data matrices are not already standardized, we standardize them. Then, by denoting $\mathcal{D} = (Y_1, ..., Y_T)$, we have the following likelihood function:

$$P(\mathcal{D}|\mathcal{M}, \boldsymbol{\Theta}) \propto \left(\prod_{t=1}^{T}|\Theta_t|^{\frac{n_t}{2}}\right)\exp\left(-\frac{1}{2}\sum_{t=1}^{T}\sum_{j=1}^{n_t}Y'_{t,j}\Theta_t Y_{t,j}\right),$$

where $Y_{t,j}$ is the j-th observation vector at time point t.

We could write the joint distribution as

$$\begin{aligned} P(\mathcal{D}, \mathcal{M}, \boldsymbol{\Theta}) \propto & \left(\prod_{t=1}^{T}|\Theta_t|^{\frac{n_t}{2}}\right)\exp\left(-\tfrac{1}{2}\sum_{t=1}^{T}\sum_{j=1}^{n_t}Y'_{t,j}\Theta_t Y_{t,j}\right) \\ & \exp\left(-\tfrac{\lambda}{2}\sum_{t=1}^{T}||\Theta_t||_1 - \tfrac{\rho}{2}\sum_{t=2}^{T}||\Theta_t - \Theta_{t-1}||_1\right). \end{aligned}$$

The objective function of the dynamic GLasso problem equals (up to a constant term) the logarithm of the joint distribution $P(\mathcal{D}, \mathcal{M}, \boldsymbol{\Theta})$.

SI.3 Derivation - Bayesian information criterion

For a given model $\mathcal{M}$, model parameter θ and data $\mathcal{D}$, the score function for model $\mathcal{M}$ is derived as

$$S(\mathcal{M}|\mathcal{D}) \approx \quad \log P(\mathcal{D}|\widehat{\theta}_{\mathrm{MAP}}, \mathcal{M}) + \log P(\widehat{\theta}_{\mathrm{MAP}}|\mathcal{M}) + \tfrac{k}{2} \log \tfrac{2\pi}{N},$$

where $\widehat{\theta}_{\mathrm{MAP}}$ is the *maximum a posteriori* estimate of θ. The terms k and N are the dimension of the parameter space and the sample size, respectively. The function $S(\mathcal{M}|\mathcal{D})$ is the score function derived from the posterior distribution of the model under log form:

$$S(\mathcal{M}|\mathcal{D}) = \log P(\mathcal{M}|\mathcal{D}) = \log \frac{P(\mathcal{M}, \mathcal{D})}{P(\mathcal{D})} = \log \frac{P(\mathcal{D}|\mathcal{M})P(\mathcal{M})}{P(\mathcal{D})}.$$

The probability $P(\mathcal{M})$ is constant under the uniform model selection assumption and the probability $P(\mathcal{D})$ is constant for all $\mathcal{D}$.

Hence,

$$S(\mathcal{M}|\mathcal{D}) \propto \log P(\mathcal{D}|\mathcal{M}),$$

and

$$P(\mathcal{D}|\mathcal{M}) = \int_\theta P(\mathcal{D}, \theta|\mathcal{M}) d\theta = \int_\theta P(\mathcal{D}|\theta, \mathcal{M}) P(\theta|\mathcal{M}) d\theta.$$

After local approximation of the parameter posterior around $\widehat{\theta}_{\mathrm{MAP}}$,

$$\begin{aligned}\log P(\theta|\mathcal{D}, \mathcal{M}) \approx &\log P(\widehat{\theta}_{\mathrm{MAP}}|\mathcal{D}, \mathcal{M}) + (\theta - \widehat{\theta}_{\mathrm{MAP}})' \frac{\partial}{\partial \theta} \log P(\theta|\mathcal{D}, \mathcal{M})|_{\theta = \widehat{\theta}_{\mathrm{MAP}}} \\ &+ \frac{1}{2} (\theta - \widehat{\theta}_{\mathrm{MAP}})'(-H)(\theta - \widehat{\theta}_{\mathrm{MAP}}),\end{aligned}$$

where H is the Fisher information matrix.

Since

$$\frac{\partial}{\partial \theta} \log P(\theta|\mathcal{D}, \mathcal{M})|_{\theta = \widehat{\theta}_{\mathrm{MAP}}} = 0.$$

We have

$$\log P(\theta|\mathcal{D}, \mathcal{M}) \approx \log P(\widehat{\theta}_{\mathrm{MAP}}|\mathcal{D}, \mathcal{M}) - \frac{1}{2} (\theta - \widehat{\theta}_{\mathrm{MAP}})' H (\theta - \widehat{\theta}_{\mathrm{MAP}}).$$

This implies

$$P(\theta|\mathcal{D}, \mathcal{M}) \approx P(\widehat{\theta}_{\mathrm{MAP}}|\mathcal{D}, \mathcal{M}) \exp \left(-\frac{1}{2} (\theta - \widehat{\theta}_{\mathrm{MAP}})' H (\theta - \widehat{\theta}_{\mathrm{MAP}}) \right).$$

Now,

$$\begin{aligned}
P(\mathcal{D}|\mathcal{M}) &= \int_\theta P(\mathcal{D},\theta|\mathcal{M})d\theta \\
&\propto \int_\theta P(\theta|\mathcal{D},\mathcal{M})d\theta \qquad\qquad \text{since } \mathcal{M} \text{ and } \mathcal{D} \text{ are fixed.} \\
&\approx \int_\theta P(\widehat{\theta}_{\text{MAP}}|\mathcal{D},\mathcal{M}) \exp\left(-\frac{1}{2}(\theta-\widehat{\theta}_{\text{MAP}})'H(\theta-\widehat{\theta}_{\text{MAP}})\right) d\theta \\
&\approx \int_\theta P(\mathcal{D}|\widehat{\theta}_{\text{MAP}},\mathcal{M})P(\widehat{\theta}_{\text{MAP}}|\mathcal{M}) \exp\left(-\frac{1}{2}(\theta-\widehat{\theta}_{\text{MAP}})'H(\theta-\widehat{\theta}_{\text{MAP}})\right) d\theta \\
&= P(\mathcal{D}|\widehat{\theta}_{\text{MAP}},\mathcal{M})P(\widehat{\theta}_{\text{MAP}}|\mathcal{M}) \int_\theta \exp\left(-\frac{1}{2}(\theta-\widehat{\theta}_{\text{MAP}})'H(\theta-\widehat{\theta}_{\text{MAP}})\right) d\theta \\
&= P(\mathcal{D}|\widehat{\theta}_{\text{MAP}},\mathcal{M})P(\widehat{\theta}_{\text{MAP}}|\mathcal{M})(2\pi)^{k/2}|H|^{-1/2}
\end{aligned}$$

where k is the number of free parameters.

The quantity $\log|H|$ can be approximated by $k\log(N)$. This approximation holds true to order $O(1/N)$ under weak assumptions.

Taking the Laplace approximation of the marginal likelihood under log form,

$$\log P(\mathcal{D}|\mathcal{M}) \approx \log P(\mathcal{D}|\widehat{\theta}_{\text{MAP}},\mathcal{M}) + \log P(\widehat{\theta}_{\text{MAP}}|\mathcal{M}) + \frac{k}{2}\log\left(\frac{2\pi}{N}\right).$$

SI.4 Normalization constant for the prior distribution

The normalization constant of the prior distribution is denoted by $c(\lambda, \rho, T, p)$. It is a function of four variables, namely, the sparsity parameter (λ), the smoothing parameter (ρ), the number of time points (T) and the number of nodes (p). This term is computed by applying iterated integration for a fixed T as follows:

$$\begin{aligned} c(\lambda, \rho, T, p) &= \frac{p(p+1)}{2} c(\lambda, \rho, T) \\ \frac{1}{c(\lambda, \rho, T)} &= \int_{x_T} \int_{x_{T-1}} ... \int_{x_1} \exp\left(-\lambda \sum_{i=1}^{T} |x_i| - \rho \sum_{i=2}^{T} |x_i - x_{i-1}|\right) dx_1\, dx_2 \ldots dx_T \\ &= \int_{x_T} \int_{x_{T-1}} \exp\left(-\lambda |x_T| - \rho |x_T - x_{T-1}|\right) C^*(\lambda, \rho, x_{T-1})\, dx_T\, dx_{T-1}, \end{aligned}$$

where $C^*(\lambda, \rho, x_{T-1})$ is the integral with respect to $x_1, x_2, \ldots, x_{T-2}$. A closed-form expression of $c(\lambda, \rho, T)$ for arbitrary T is hard to find; however it can be computed iteratively as mentioned above. Below are some examples:

$$\begin{aligned} c(\lambda, \rho, T=2) &= \frac{\lambda(\lambda+\rho)^2}{2(2\lambda+\rho)} \\ c(\lambda, \rho, T=3) &= \frac{3\lambda(\lambda+\rho)^2(2\lambda+\rho)(\lambda+2\rho)}{8(6\lambda^2+9\lambda\rho+2\rho^2)} \\ c(\lambda, \rho, T=4) &= \frac{\lambda(\lambda+\rho)^3(2\lambda+\rho)^2(\lambda+2\rho)^2(3\lambda+\rho)}{4(48\lambda^5+184\lambda^4\rho+256\lambda^3\rho^2+161\lambda^2\rho^{+}44\lambda\rho^4+4\rho^5)}. \end{aligned}$$

SI.5 Algorithm - dynamic graphical lasso

In this section, we describe an algorithm to estimate precision matrices that are smooth over time.

Algorithm 1 Dynamic Sparse Covariance Estimation using Standardized Data

INPUT: Raw datasets $\mathcal{D}_t, t = 1, \ldots, T$, sample size $n_t, t = 1, \ldots, T$, topology selection threshold $\tau(\geq 0)$, $M = 10^{15}$,

OUTPUT: Estimated precision matrices $\widehat{P}_t, t = 1, \ldots, T$

Standardize datasets $\mathcal{D}_t$ to $\mathcal{D}'_t, \forall t$

Compute empirical covariance matrices S_t from datasets $\mathcal{D}'_t, \forall t$.

Calculate maximum value of regularized parameters

$$\begin{aligned} \textbf{Sparsity parameter, } \lambda^* &= \max_{t,i,j,i\neq j} (n_t S_{t,ij}) \\ \textbf{Smoothing parameter, } \rho^* &= \max_t (n_t, n_{t-1}) \max_{t,i,j,i\neq j} (S_{t,ij} - S_{t-1,ij}) \end{aligned}$$

Create set of regularized parameter by selecting uniformly discrete values over the interval $[\eta\lambda^*, \lambda^*]$ and $[\eta\rho^*, \rho^*]$, where $\eta \in (0, 1)$.

for all λ, ρ **do**

  Solve optimization problem

$$\begin{aligned} (Y_1, \ldots, Y_T) = \arg \max_{\Theta_i \succ 0, \forall i} & \sum_{i=1}^{T} \frac{n_i}{2} [\log(\det(\Theta_i)) - \mathrm{Tr}(S_i \Theta_i)] \\ & - \frac{\lambda}{2} \sum_{i=1}^{T} ||\Theta_i||_1 - \frac{\rho}{2} \sum_{i=2}^{T} ||\Theta_i - \Theta_{i-1}||_1 \end{aligned}$$

  Select topology from $(Y_1, \ldots, Y_T)$ with given threshold τ

  for all i, j **do**

$$\lambda^*_{l,ij} \leftarrow \begin{cases} M, & \text{if } |Y_{l,ij}| \leq \tau \\ 0, & \text{else} \end{cases}$$

  end for

  Solve optimization problem for given topology

$$\begin{aligned} (\widehat{X}_1, \ldots, \widehat{X}_T) = \arg \max_{\Theta_i \succ 0, \forall i} & \sum_{i=1}^{T} \frac{n_i}{2} [\log(\det(\Theta_i)) - \mathrm{Tr}(S_i \Theta_i)] \\ & - \sum_{i=1}^{T} \frac{1}{2} ||\lambda^*_i \circ \Theta_i||_1 - \frac{\rho}{2} \sum_{i=2}^{T} ||\Theta_i - \Theta_{i-1}||_1 \end{aligned}$$

12: Calculate BIC

$$
\begin{aligned}
BIC(\lambda, \rho) = & \sum_{i=1}^{T} -\frac{n_i}{2} \left[\log(\det(\hat{X}_i)) - \mathrm{Tr}(S_i \hat{X}_i) \right] \\
& + \frac{\lambda}{2} \sum_{i=1}^{T} ||\hat{X}_i||_1 + \frac{\rho}{2} \sum_{i=2}^{T} ||\hat{X}_i - \hat{X}_{i-1}||_1 \\
& + \sum_{i=1}^{T} \left[\frac{k_i}{2} \log \frac{n_i}{2\pi} \right] - \log(c(\lambda, \rho, T, p)),
\end{aligned}
$$

where $c(\lambda, \rho, T, p)$ is a constant factor of the prior distribution.

13: **end for**

14: Choose $(\hat{\lambda}, \hat{\rho})$ such that $BIC(\hat{\lambda}, \hat{\rho}) \leq BIC(\lambda, \rho), \forall (\lambda, \rho)$

15: **if** $(\hat{\lambda}, \hat{\rho})$ is at border points **then**

16: Extend the set of parameters around border points.

17: Go to step 5

18: **end if**

19: Assign $\widehat{X}_i$ to $\widehat{\Theta}_i, \forall i$

SI.6 Algorithm implementation

We used the Matlab package `YALMIP` and `SDPT3` solver to find the optimal solution of our optimization problem[1,2]. `YALMIP` is a Matlab modeling language to solve convex optimization problems. `SDPT3` solver is specially designed to solve conic optimization problems whose constraint cone is a product of semidefinite cones, second-order cones and nonnegative orthants. It uses a predictor-corrector primal-dual infeasible path following algorithm, with either the HKM[3,4,5] or the NT[6] search direction. We have also used a recently developed solver package, `SCS` (Splitting Conic Solver), that solves the convex cone problems using the alternating direction method of multipliers (ADMM)[7]. The estimated precision matrices using both solvers converge to the same optimal solution.

[1] Lofberg J. YALMIP: a toolbox for modeling and optimization in MATLAB. *IEEE International Symposium, in Computer Aided Control Systems Design*, 284-289 (2004)

[2] Toh K.C. *et al.* SDPT3 - A Matlab software package for semidefinite programming. *Optimization Methods and Software*, **11**, 545-581 (1999)

[3] Helmberg C. *et al.* An interior-point method for semidefinite programming. *SIAM Journal on Optimization*, **6**, 342-361 (1996)

[4] Kojima M. *et al.* Interior-point methods for the monotone linear complementarity problem in symmetric matrices. *SIAM Journal on Optimization* **7**, 86-125 (1997)

[5] Monteiro R.D.C. Primal-dual path-following algorithms for semidefinite programming. *SIAM Journal on Optimization* **7**, 663-678 (1997)

[6] Nesterov Y.E. & Todd M.J. Self-scaled barriers and interior-point methods in convex programming. *Math. Oper. Res.* **22**, 1-42 (1997)

[7] O'donoghue B. Conic optimization via operator splitting and homogeneous self-dual embedding. *Journal of Optimization Theory and Applications* **169(3)**, 1042-1068 (2016)

SI.7 Validation of model-selection algorithm

We applied the Bayesian information criterion (BIC) as a model score function to estimate the sparsity and smoothing parameters. The BIC approximates the posterior density of a model conditional on the data in negative logarithm scale. The model with the lowest BIC score is selected.

We estimated the joint posterior density of the regularization parameters, i.e., the sparsity and smoothing parameters, using MCMC (Markov Chain Monte Carlo) and used it to validate the BIC score function. Therefore, we simulated a time-series dataset with 5 variables over 3 time points. We followed the paper by Zakaria *et al.*[8] to estimate the joint posterior density of the regularization parameters. We selected 20,000 MCMC samples after 30,000 burn-ins to obtain the joint posterior distribution. The performance of the regularization parameters selected using BIC is illustrated in fig. S.7.1.

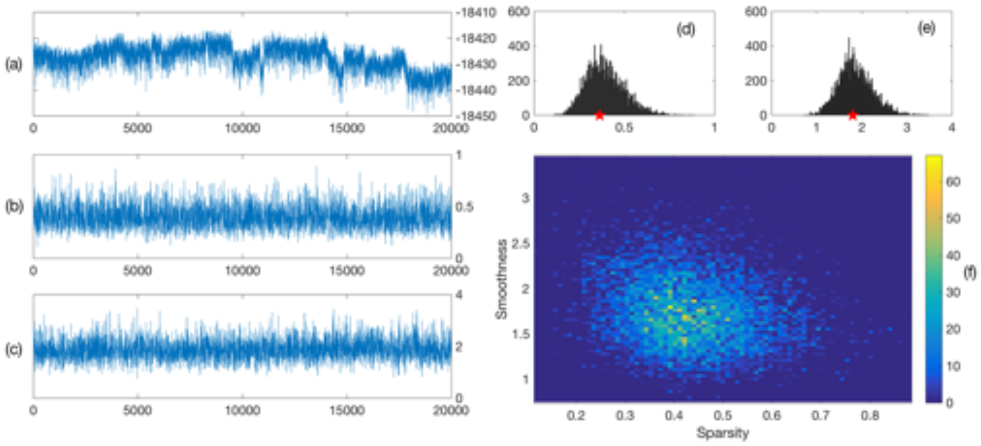

Figure S.7.1: Posterior density $f(\lambda, \rho|\mathcal{D})$ estimation using MCMC: The figure shows the results obtained from an MCMC of length 20,000 after a burn-in period of length 30,000. **(a)** log likelihood plot of the sample data, **(b)** trace plot for the sparsity parameter (λ), **(c)** trace plot for the smoothing parameter (ρ), **(d)** posterior distribution of the sparsity parameter shown as histogram, **(e)** posterior distribution of the smoothing parameter shown as histogram and **(f)** joint posterior density plot of both regularization parameters. The red markers in sub-figures (d) and (e) show the estimated regularization parameters using the variant of BIC score function. We verified the convergence of the Markov chain using diagnostic approaches, such as the auto-correlation function and the potential scale reduction factor.

[8] Zakaria K.S. *et al.* The Bayesian covariance lasso. *Statistics and its Interface* **6**, 243-259 (2013)

SI.8 Algorithm - model posterior estimation using Markov Chain Monte Carlo

This section explains the algorithm to estimate the posterior distribution of the regularization parameters and the precision matrices using MCMC (Markov Chain Monte Carlo).

Algorithm 2 Model posterior sampling scheme

INPUT: Time-series dataset $\mathcal{D}$; log-likelihood function $L(\lambda, \rho, P_1, P_2, \ldots, P_T|\mathcal{D})$; the number of variables p ; sample precision matrices $\tilde{P}_i, t = 1, \ldots, T$; MCMC iteration M

OUTPUT: Sampled set $(\lambda, \rho, P_1, P_2, \ldots, P_T)$

Compute variance for the proposal distribution of diagonal elements of precision matrices

$$V_{t,ii} = \Big(\frac{|\tilde{P}_t|}{|\tilde{P}_{t,ii}|}\Big)^2 \forall t = 1, 2, \ldots, T; i = 1, \ldots, p$$

where $\tilde{P}_{t,ii}$ is the sub-matrix from precision matrix $\tilde{P}_t$ after removing i^{th} row and column.

Compute variance for the proposal distribution of off-diagonal vectors of precision matrices

$$V_{t,i} = \Big(\frac{-2}{V_{t,ii}}\tilde{P}^{-1}_{t,ii}\tilde{P}_{t,i}\tilde{P}'_{t,i}\tilde{P}^{-1}_{t,ii} + \frac{2}{V_{t,ii}}\tilde{P}^{-1}_{t,ii}\Big)^{-1} \forall t = 1, 2, \ldots, T; i = 1, \ldots, p$$

where $\tilde{P}_{t,i}$ is the ith off-diagonal vector of the sample precision matrix $\tilde{P}_t$.

for all Iteration = 1 to M **do**

{**Sampling Sparsity Parameter**}
Generate λ_{new} from gamma distribution $G(\lambda|a_{old}, b_{old})$ with mean λ_{old} and variance $V_\lambda(\lambda_{old}, \rho_{old})$

Calculate forward probability $G(\lambda_{new}|a_{old}, b_{old})$ and backward probability $G(\lambda_{old}|a_{new}, b_{new})$.

Calculate Hastings Ratio

$$H_\lambda = \frac{\exp(L(\lambda_{new}, \rho_{old}, P_{1,old}, P_{2,old}, \ldots, P_{T,old}|\mathcal{D}))G(\lambda_{old}|a_{new}, b_{new})}{\exp(L(\lambda_{old}, \rho_{old}, P_{1,old}, P_{2,old}, \ldots, P_{T,old}|\mathcal{D}))G(\lambda_{new}|a_{old}, b_{old})}$$

if $H_\lambda > U(0, 1)$ **then**

Accept λ_{new} as the new value of λ_{old}

else

Reject λ_{new} and keep λ_{old} as same.

end if

{**Sampling Smoothing Parameter**}
Generate ρ_{new} from gamma distribution $G(\rho|a_{old}, b_{old})$ with mean ρ_{old} and variance $V_\rho(\lambda_{old}, \rho_{old})$ where

$$V_\rho(x, y) = V_\lambda(x, y)$$

Calculate forward probability $G(\rho_{new}|a_{old}, b_{old})$ and backward probability $G(\rho_{old}|a_{new}, b_{new})$.

Calculate Hastings Ratio

$$H_\rho = \frac{\exp(L(\lambda_{old}, \rho_{new}, P_{1,old}, P_{2,old}, \ldots, P_{T,old}|\mathcal{D}))G(\rho_{old}|a_{new}, b_{new})}{\exp(L(\lambda_{old}, \rho_{old}, P_{1,old}, P_{2,old}, \ldots, P_{T,old}|\mathcal{D}))G(\rho_{new}|a_{old}, b_{old})}$$

if $H_\rho > U(0,1)$ **then**
  Accept ρ_{new} as the new value of ρ_{old}
else
  Reject ρ_{new} and keep ρ_{old} as same.
end if
for all $t = 1, \ldots, T$ and $i = 1, \ldots, p$ **do**
  {**Sampling Diagonal Elements of Precision Matrices**}
  Draw m independent samples, $d_1, d_2, \ldots, d_m$ from the proposal distribution $\mathcal{N}(P_{t,old,ii}, V_{t,ii})$
  Select d_j from the set $(d_1, d_2, \ldots, d_m)$ with probability proportional to $\exp(L(\lambda_{old}, \rho_{old}, P_t(d_j)|\mathcal{D}))$. Denote the selected sample as d^*.
  Draw $d_1^*, d_2^*, \ldots, d_{m-1}^*$ from $\mathcal{N}(d^*, V_{t,ii})$. Take d_m^* as $P_{t,old,ii}$
  Replace $P_{t,old,ii}$ by d^* with probability

$$\min\{1, \frac{\sum_{j=1}^m \exp(L(\lambda_{old}, \rho_{old}, P_t(d_j)|\mathcal{D}))}{\sum_{j=1}^m \exp(L(\lambda_{old}, \rho_{old}, P_t(d_j^*)|\mathcal{D}))}\}$$

  {**Sampling Off-diagonal Elements of Precision Matrices**}
  Draw m independent vectors, $d_1, d_2, \ldots, d_m$ from the proposal distribution $\mathcal{N}(P_{t,old,i}, V_{t,i})$
  Select d_j from the set $(d_1, d_2, \ldots, d_m)$ with probability proportional to $\exp(L(\lambda_{old}, \rho_{old}, P_t(d_j)|\mathcal{D}))$. Denote the selected sample as d^*.
  Draw $d_1^*, d_2^*, \ldots, d_{m-1}^*$ from $\mathcal{N}(d^*, V_{t,i})$. Take d_m^* as $P_{t,old,i}$
  Replace $P_{t,old,i}$ by d^* with probability

$$\min\{1, \frac{\sum_{j=1}^m \exp(L(\lambda_{old}, \rho_{old}, P_t(d_j)|\mathcal{D}))}{\sum_{j=1}^m \exp(L(\lambda_{old}, \rho_{old}, P_t(d_j^*)|\mathcal{D}))}\}$$

end for
end for

SI.9 Synthetic data results

SI.9.1 Synthetic data generation

For the purpose of illustration of our method, we randomly generated a directed graph with sparsity level 85%. The elements of the weighted adjacency matrix W corresponding to the directed graph are selected uniformly over the range $(-1,-0.2]\cup[0.2,1)$. The nonzero entries W_{ij} can be interpreted as a directed edge from X_j to X_i with weight W_{ij}. We transformed the weighted adjacency matrix W to the precision matrix using the following transformation,

$$\Theta_1 = (I-W)^T(I-W), \tag{1}$$

where I is the identity matrix.

We successively added randomly generated positive definite matrices to the precision matrix in (1) to get the precision matrices at other time points. Finally, we simulated data from these precision matrices. The synthetic networks were created by placing an edge between two nodes if the corresponding entry in the precision matrix is nonzero. The sparsity level was set between 75% to 80%. (see Figure S.9.1 and table S.9.1)

**Time point**	**Sparsity level**	**New edges count**	**% of new edges**	**Count of vanished edges**	**% of vanished edges**
1	77.01%	-	-	-	-
2	79.54%	(1/89)	1.12%	(12/346)	3.47%
3	78.16%	(17/95)	17.89%	(11/340)	3.24%
4	77.01%	(13/100)	13.00%	(8/335)	2.39%
5	76.55%	(16/102)	15.69%	(14/333)	4.20%
6	75.63%	(6/106)	5.66%	(2/329)	0.61%

Table S.9.1: Synthetic graph summary. The table shows the sparsity levels of synthetic undirected networks at different time points.

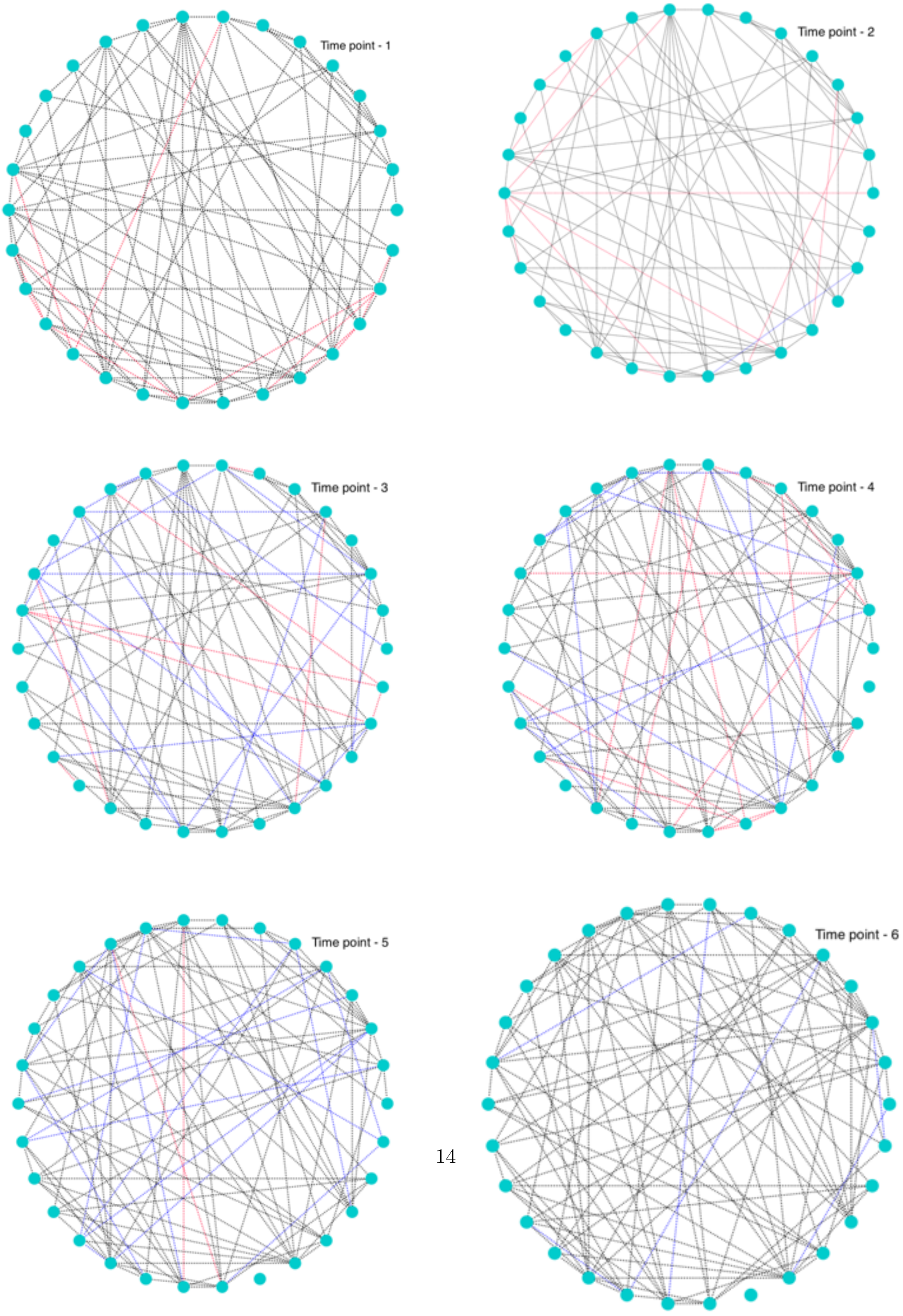

Figure S.9.1: Synthetic networks. True conditional independence graphs under *in-silico* study for all time points. The unconnected nodes are independent of each other conditionally on the rest. The set of the edges that are going to vanish in the next time point are shown in red. The set of edges in blue represents new edges.

SI.9.2 Cross validation v/s Bayesian information criterion

The dynamic graphical lasso problem is highly dependent on the regularization parameters,i.e., sparsity and smoothing parameters. There are two different approaches available in the statistical literature to estimate the regularization parameters - (a) Cross validation (CV), (b) Bayesian information criterion (BIC). We implemented both model selection schemes to estimate sparse precision matrices over time by setting the smoothing parameter to zero. The precision matrices estimated by CV showed large number of non-zero entries. BIC performed better with respect to the sparsity of the precision matrices. The BIC score function penalizes the number of non-zero elements in the precision matrices. Therefore, the estimated precision matrices based on BIC were sparser (see Fig. S.9.2).

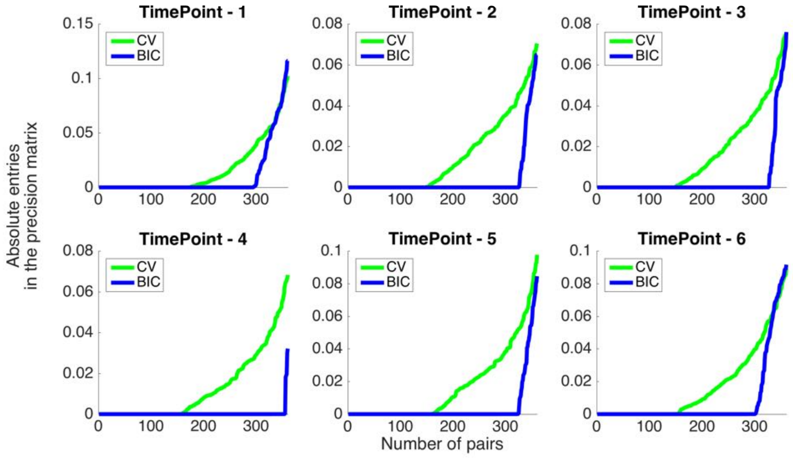

Figure S.9.2: Comparison between cross validation (CV) and Bayesian information criterion (BIC). The figures show the absolute entries of the estimated precision matrices in ascending order. BIC clearly yields greater sparsity compared to CV.

Time Point	Glasso (CV)	Glasso (BIC)
1	159.2 ± 16.27	301.5 ± 9.49
2	173.7 ± 15.08	316.6 ± 14.2
3	176.6 ± 16.14	327.1 ± 14.09
4	175.4 ± 15.14	339.9 ± 17.61
5	168.7 ± 11.93	325.3 ± 10.55
6	174.6 ± 11.44	312.4 ± 9.023

Table S.9.2: Achieved sparsity level for CV and BIC. The table shows the number of pairs of entries of the precision matrices that are absolutely less than 10^{-3}. The mean number of pairs is calculated using 20 datasets. These values clearly show that the estimated graphs using BIC are sparser than the estimated graphs using cross validation. CV favors a model with greater predictive power.

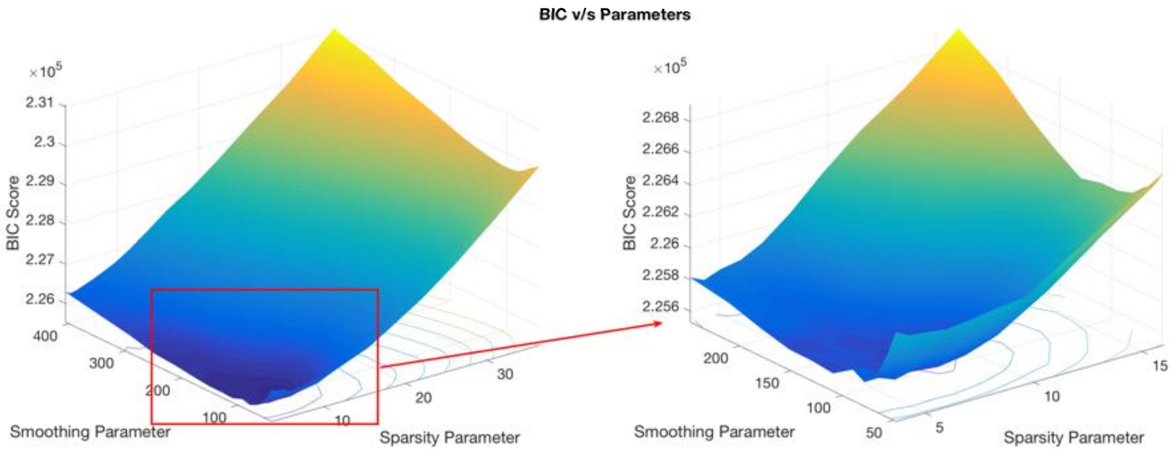

Figure S.9.3: Model selection using BIC. The figure plots BIC scores against the regularization parameters. BIC score approximates the *maximum a posteriori* (MAP) of the model in the negative logarithmic scale. We select both parameters with the lowest BIC score. For a particular dataset, the estimated sparsity and smoothing parameters are 8.3051 and 109.1782 respectively.

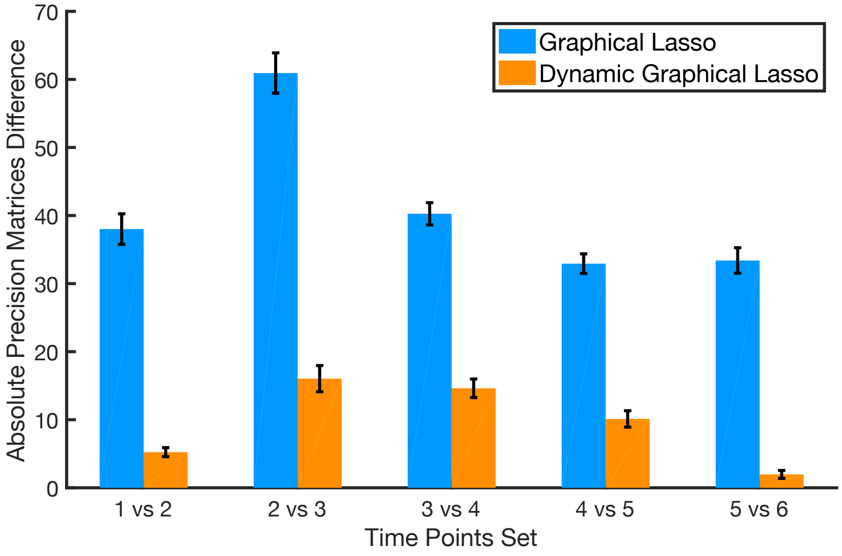

Figure S.9.4: Comparison between GLasso and dynamic GLasso by checking the effect of smoothing parameter. The bar plot shows the estimated structural variation over consecutive time points. The blue and orange bars are corresponding to the models GLasso and dynamic GLasso respectively. The mean structural variation is calculated using 20 distinct time-series datasets. Dynamic GLasso achieves lower structural variation due to the additional regularization parameter.

SI.9.3 Performance over noise strength

We evaluated the performance of the models for different noise (error) strengths for simulated data. We added correlated Gaussian noise to the simulated data with varying strength from 0 to 10. The dynamic GLasso performed better than GLasso. The model dynamic GLasso estimates networks consistently as the strength of the noise decreases (see the fig. S.9.5). The mean AUROC decreases with increase in the noise strength as expected.

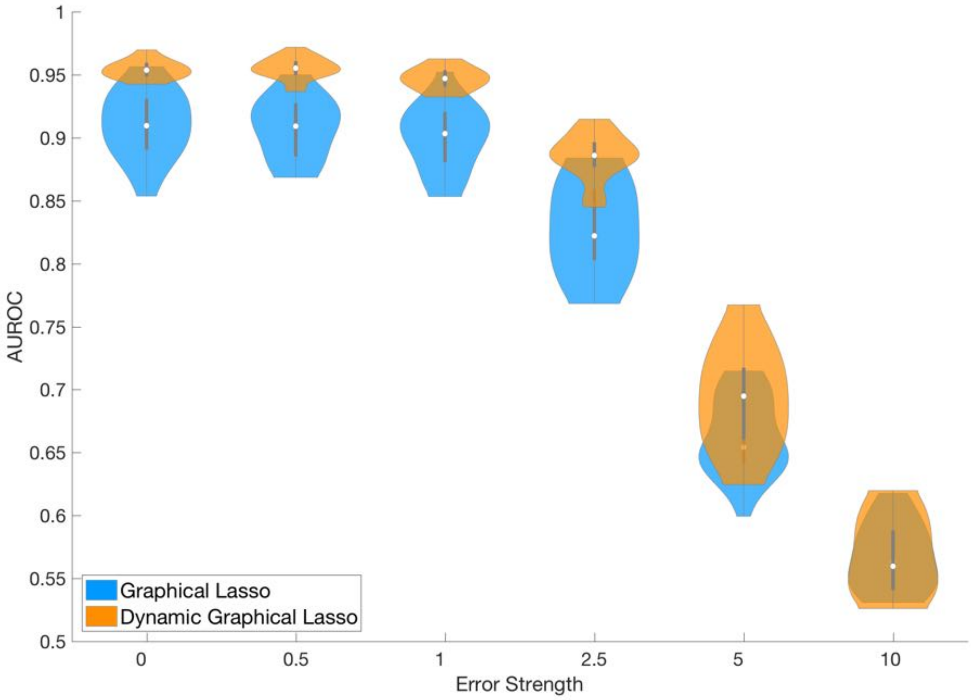

Figure S.9.5: Effect of the noise strength on the performance of the models: The plot shows the performance of the model dynamic GLasso over the static model GLasso by varying the strength of error matrix under *in-silico* study. The plots represent the kernel density estimate of AUROCs calculated using 5 datasets. The performance of dynamic GLasso inproves as the strength of noise decreases.

SI.10 Dynamic network reconstruction using block sparsity

The model dynamic graphical lasso maximizes the L_1-penalized log-likelihood of the time-series dataset. Two penalization terms are introduced in the objective function to impose sparsity and smoothness on the estimated networks. The structural variation is defined as the sum of absolute differences between the elements of two consecutive precision matrices.

We evaluated the performance of the model after replacing the smoothing penalization by block sparsity[9]. In this approach, we considered the estimated precision matrices form a block vector of size T, where T is total number of time points. We applied L_2-norm to all block vectors to ensure smoothness over the entries of the precision matrices. The block L_2-norm penalization encourages a similar pattern of sparsity across time. We also applied L_1-norm to individual precision matrices to enforce additional sparsity.

We could write the model with block sparsity mathematically as:

$$\begin{aligned}(\widehat{\Theta}_1, \ldots, \widehat{\Theta}_T) &= \arg\max_{\Theta_t \succ 0, \forall t} \sum_{t=1}^{T} \frac{n_t}{2}[\log(\det(\Theta_t)) - \mathrm{Tr}(S_t\Theta_t)] \\ &\quad -\frac{\lambda}{2}\sum_{t=1}^{T} ||\Theta_t||_1 - \frac{\rho}{2}\sum_{i,j=1}^{p} ||(\Theta_{(1,ij)}, \ldots, \Theta_{(T,ij)})||_2 \end{aligned} \qquad (2)$$

where λ and ρ denote sparsity and smoothing parameters respectively. The total number of time points is denoted by T. The standardized sample covariance matrix and the sample size at time point t are denoted by S_t and n_t respectively. The (i, j)-th element of the precision matrix Θ_t at time point t is denoted by $\Theta_{(t,ij)}$.

Under *in silico* study, we optimized the objective function (2) with respect to the simulated datasets (see section SI.9 for more on data preparation). We used ROC (Receiver Operating Characteristic) curve and AUROC (Area Under ROC) as statistical tools to compare predicted networks with the true ones. Figure S.10.1 and S.10.2 show that the area under the ROC curve for the model with block sparsity is lower than the model dynamic graphical lasso. We also compared both models separately for each time points using mean ROC curve calculated using 20 time-series datasets (see the fig. S.10.1). The heat map S.10.3 shows the entries of estimated partial correlations from both models. The model dynamic GLasso achieved greater sparsity. The sample variance of the estimted partial correlations is smaller for dynamic GLasso compared to the model with block sparsity.

[9] Eldar Y.C. *et al.* Block-sparsity: Coherence and efficient recovery. *IEEE International Conference on Acoustics, Speech and Signal Processing*, 2885-2888 (2009)

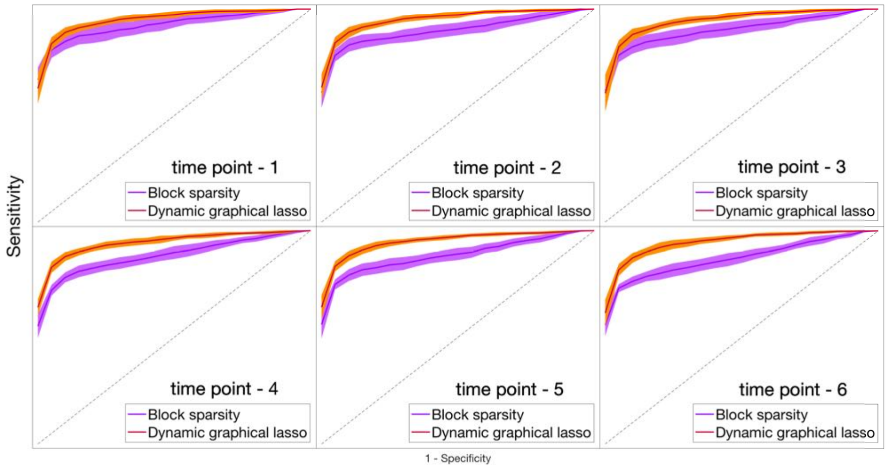

Figure S.10.1: Comparison of dynamic GLasso and block sparsity using ROC curves: The ROC curves in orange and those in violet correspond to dynamic GLasso and the model with block sparsity respectively. The mean ROC curves are estimated using 20 time-series datasets and the shaded regions correspond to respective standard deviations. The performance of the model dynamic GLasso is better than the model with block sparsity.

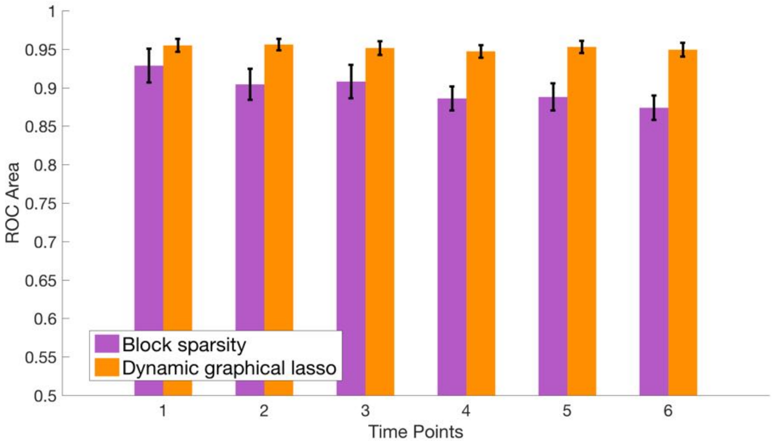

Figure S.10.2: Performance evaluation between dynamic GLasso and block sparsity with AUROCs: The vertical bars correspond to the area under ROC (AUROC) with respective standard deviation. Dynamic GLasso achieves not only higher AUROCs, but also lower standard deviations.

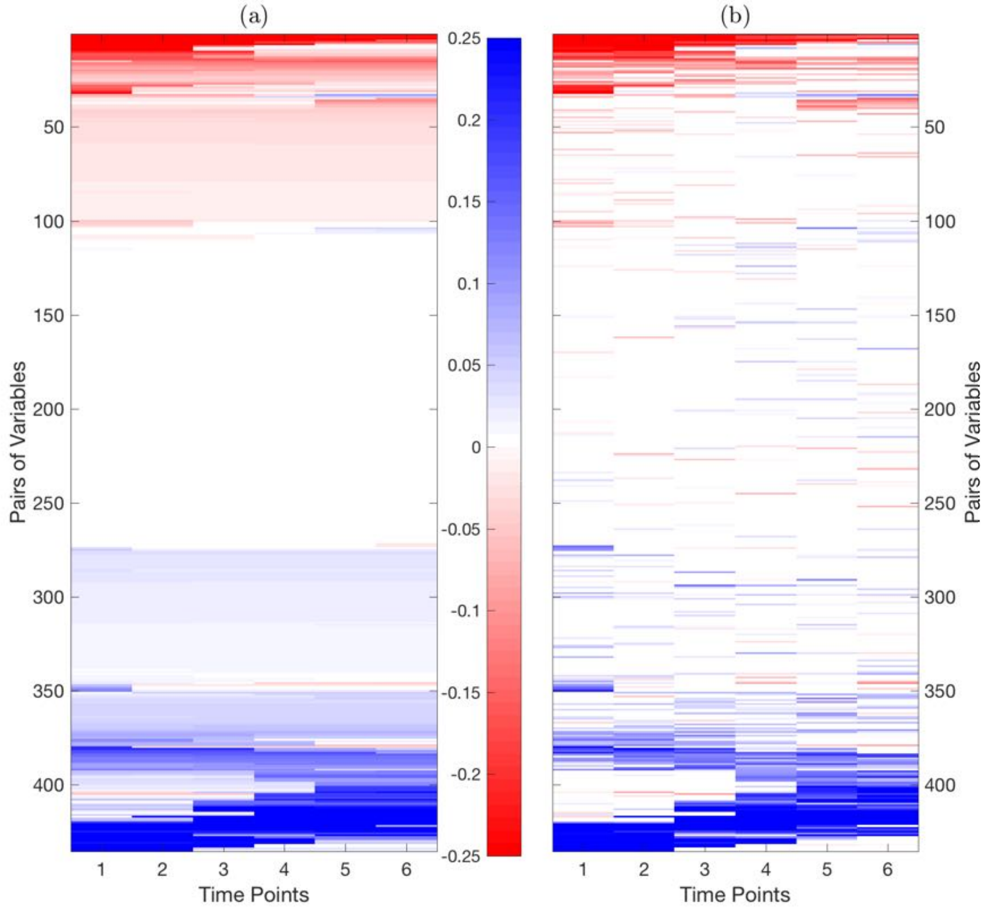

Figure S.10.3: Performance evaluation between dynamic GLasso and block sparsity using partial correlations: The figure shows the heat-map of estimated partial correlation using two different penalization constraints. **(a)** Smoothing penalization with L1-norm, Penalty $= \rho \sum_{i=2}^{T} ||\Theta_i - \Theta_{i-1}||_1$, **(b)** Smoothing penalization using block sparsity, Penalty $= \rho \sum_{i=1}^{p} \sum_{j=1}^{p} ||(\Theta_{1,ij}, \ldots, \Theta_{T,ij})||_2$. The estimated partial correlation using L1-norm penalization has low structural variation over time. The model with block sparsity estimates partial correlation with high sample variance.

SI.11 Biological data summary

SI.11.1 Fold change

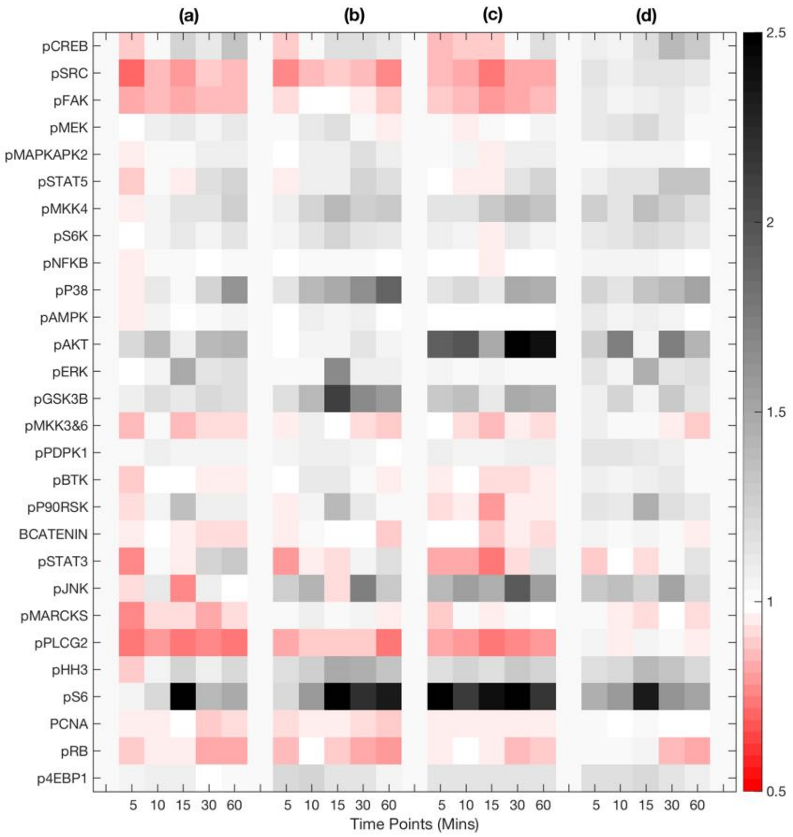

Figure S.11.1: Relative time-course protein abundance quantification. The figure shows the abundance level of phospho-proteins relative to control (time point 0 minute) from time-course experiments. The heat-map is divided into four groups based on inhibition conditions. **(a)** No Inhibition, **(b)** PI3K Inhibition, **(c)** MEK Inhibition, **(d)** AMPK Inhibition. All experiments were done with THP1 cell line using mass cytometry at single cell resolution. Cells were incubated with IFNγ for a time course of 0, 5, 10, 15, 30 and 60 minutes. Inhibitions of PI3K, MEK and AMPK reduce the phosphorylation level of AKT, ERK and FAK respectively.

SI.11.2 Results from re-constructed IFNγ stimulated networks

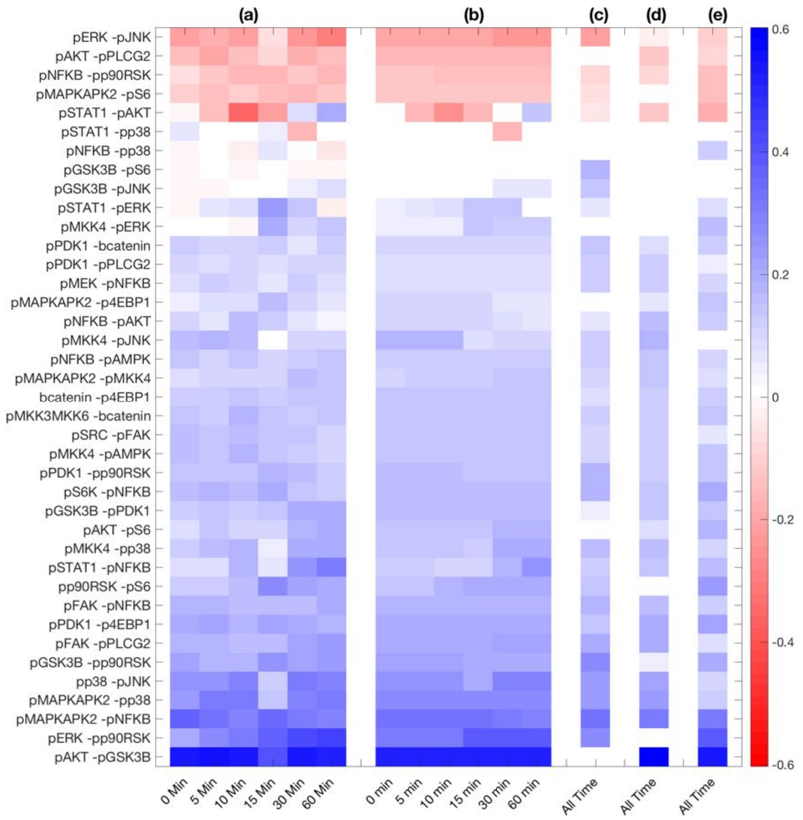

Figure S.11.2: Heatmap of estimated partial correlation under biological study. The figure shows the comparative study between the two statistical methods graphical lasso and dynamic graphical lasso. The heatmap shows the estimated partial correlation of the selected protein-pairs. We selected top 20 protein-pairs at each time point based on estimated partial correlation. **(a)** Partial correlation estimated under GLasso with "no inhibition", **(b)** Partial correlation estimated under dynamic GLasso with "no inhibition", **(c)** Estimated partial correlation from PI3K inhibited dataset, **(d)** Estimated partial correlation from MEK inhibited dataset, **(e)** Estimated partial correlation from AMPK inhibited dataset. The estimated partial correlation with graphical lasso has high sample variance over time.

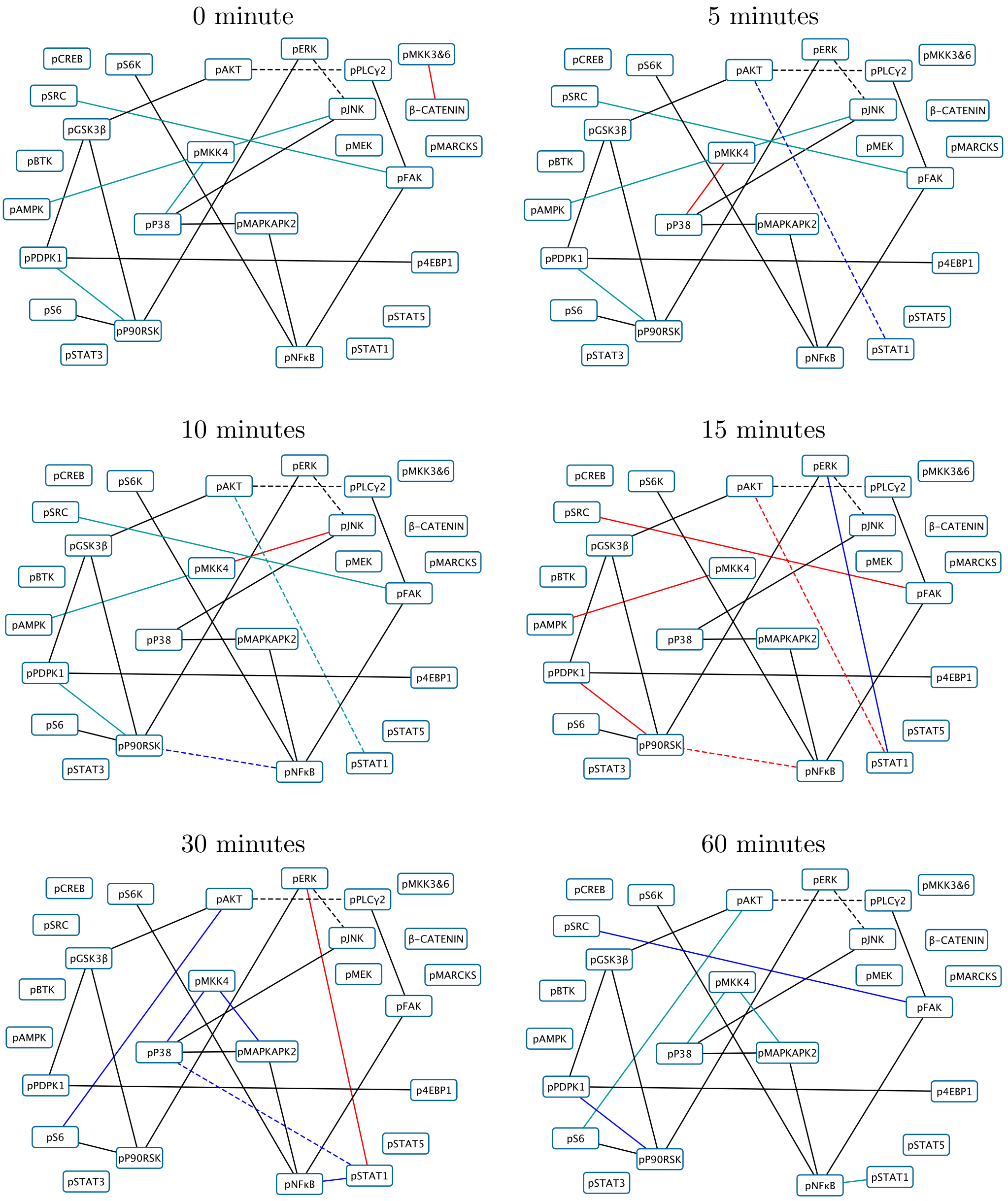

Figure S.11.3: Biological network reconstruction under control experiment. The figure shows the reconstructed conditional independence graphs from the estimated precision matrices for all time points using dynamic graphical lasso. We selected top 20 pairs with high partial correlation in absolute value. The solid and dotted edges denote the conditional dependence relationship with positive and negative partial correlation respectively. The stable edges are denoted in black. The edges that are going to vanish in the next time point are shown in red. The edges in blue represent new edges that did not appear in the previous time point. The edges in sea green are the stable edges over consecutive time points.

0 minute

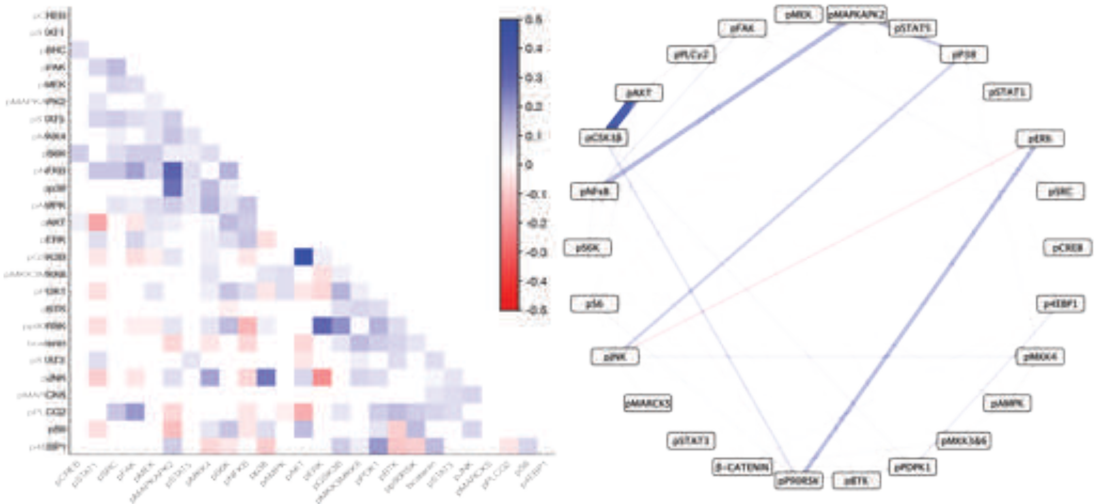

5 minutes

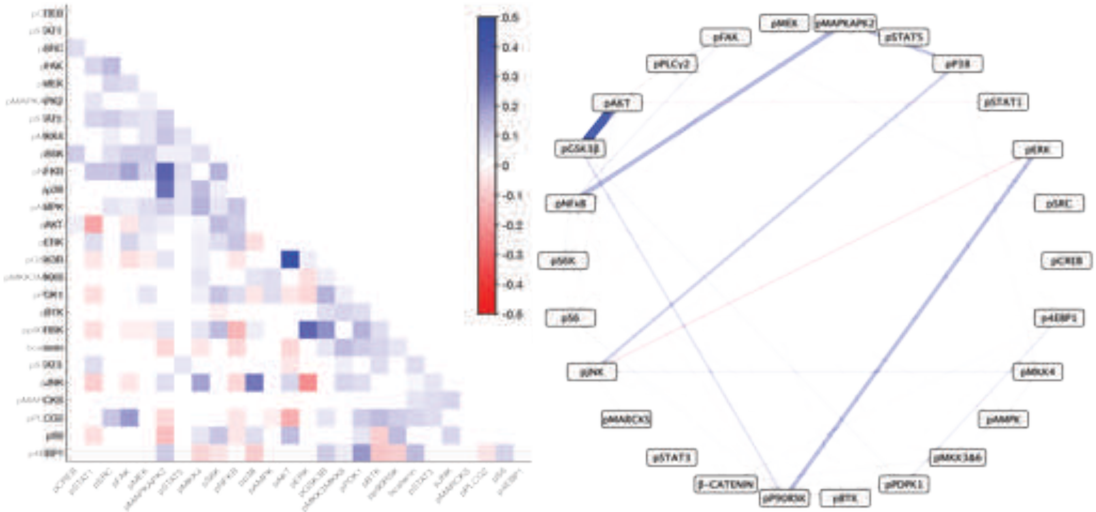

Figure S.11.4: Estimated partial correlation matrices from the control experiment (Part A). The figure shows the estimated partial correlations at time points for 0 and 5 minutes using dynamic GLasso. The thickness of the edges in the conditional independence graphs are set proportionally to the strength of partial correlations.

10 minute

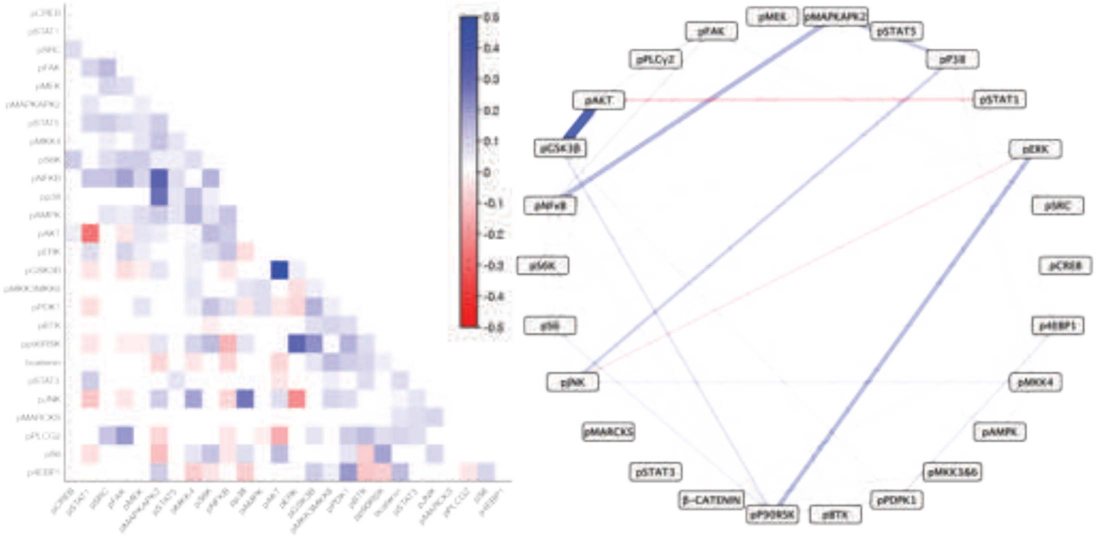

15 minutes

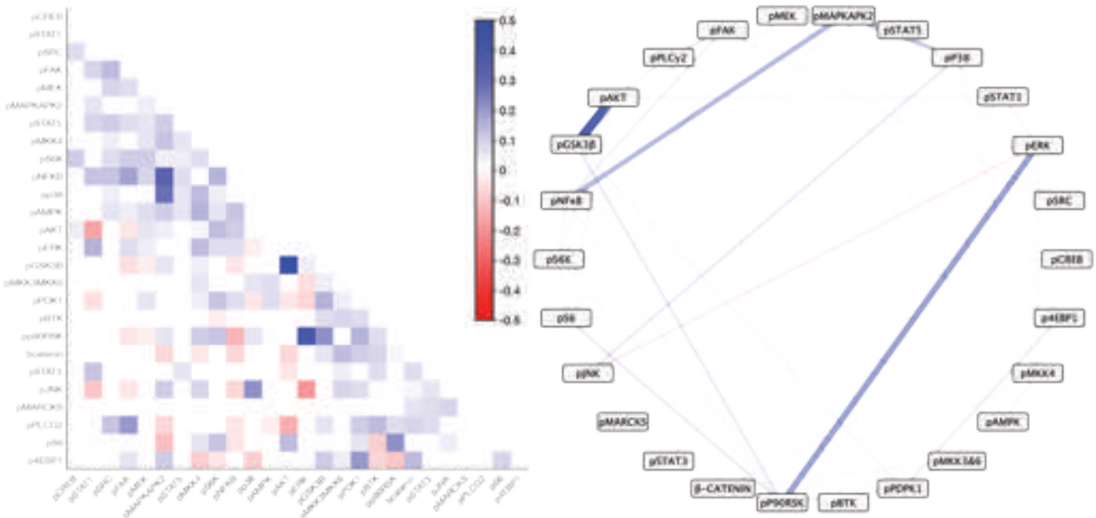

Figure S.11.5: Estimated partial correlation matrices from the control experiment (Part B). The figure shows the estimated partial correlations at time points for 10 and 15 minutes using dynamic GLasso. The thickness of the edges in the conditional independence graphs are set proportionally to the strength of partial correlations.

30 minutes

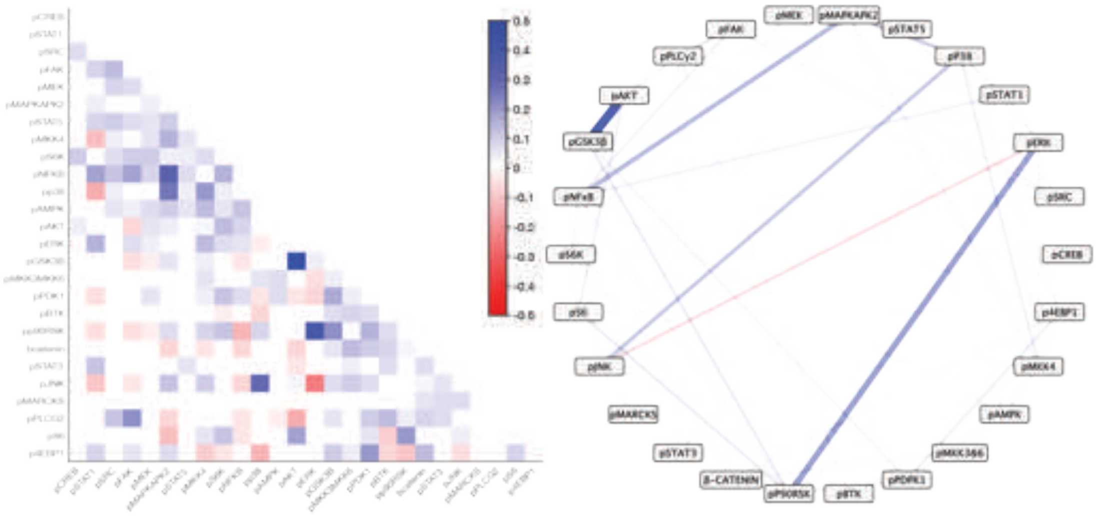

60 minutes

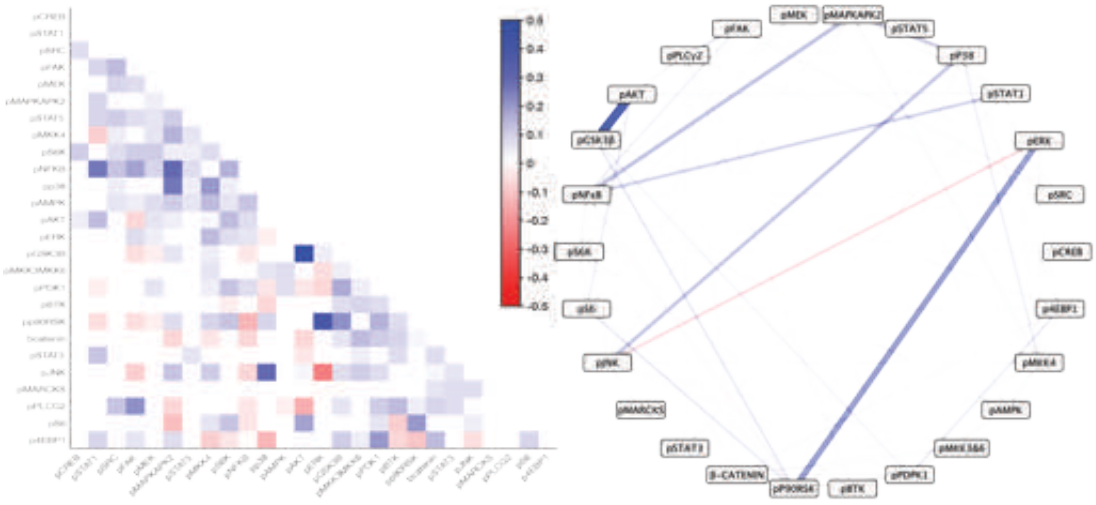

Figure S.11.6: Estimated partial correlation matrices from the control experiment (Part C). The figure shows the estimated partial correlations at time points for 30 and 60 minutes using dynamic GLasso. The thickness of the edges in the conditional independence graphs are set proportionally to the strength of partial correlations.

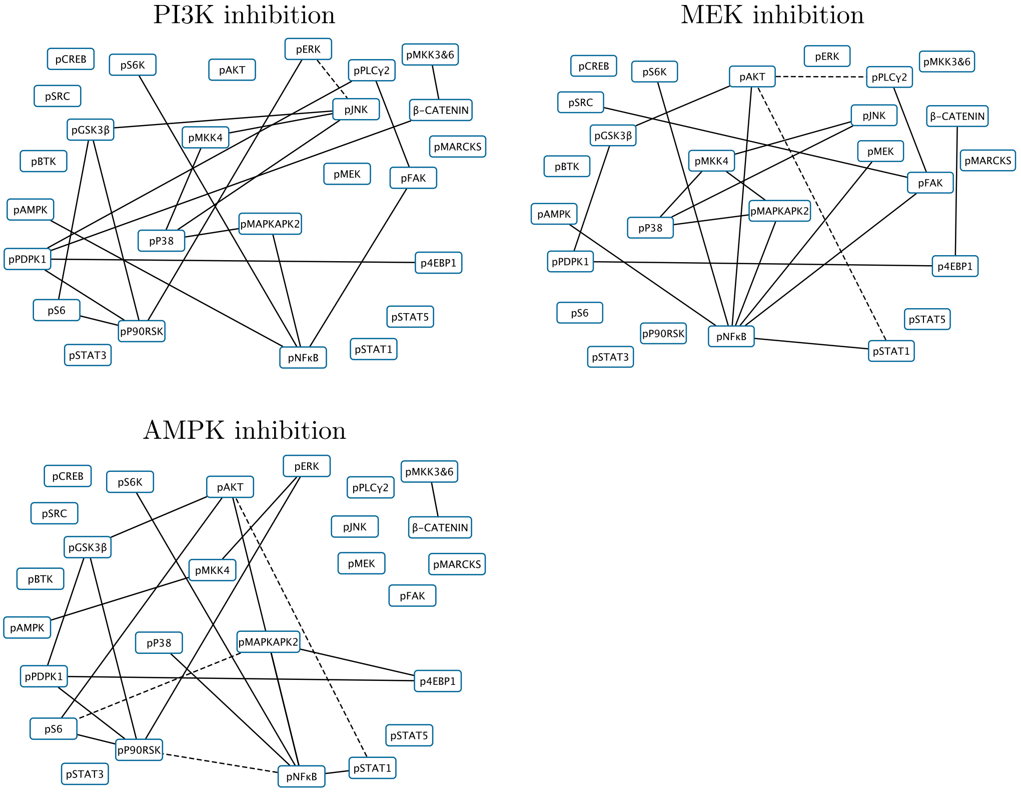

Figure S.11.7: Biological network reconstruction for inhibited experiments. The estimated undirected networks based on dynamic GLasso are same for all time points. The solid and dotted undirected edges indicate the relationship with positive and negative partial correlations respectively.

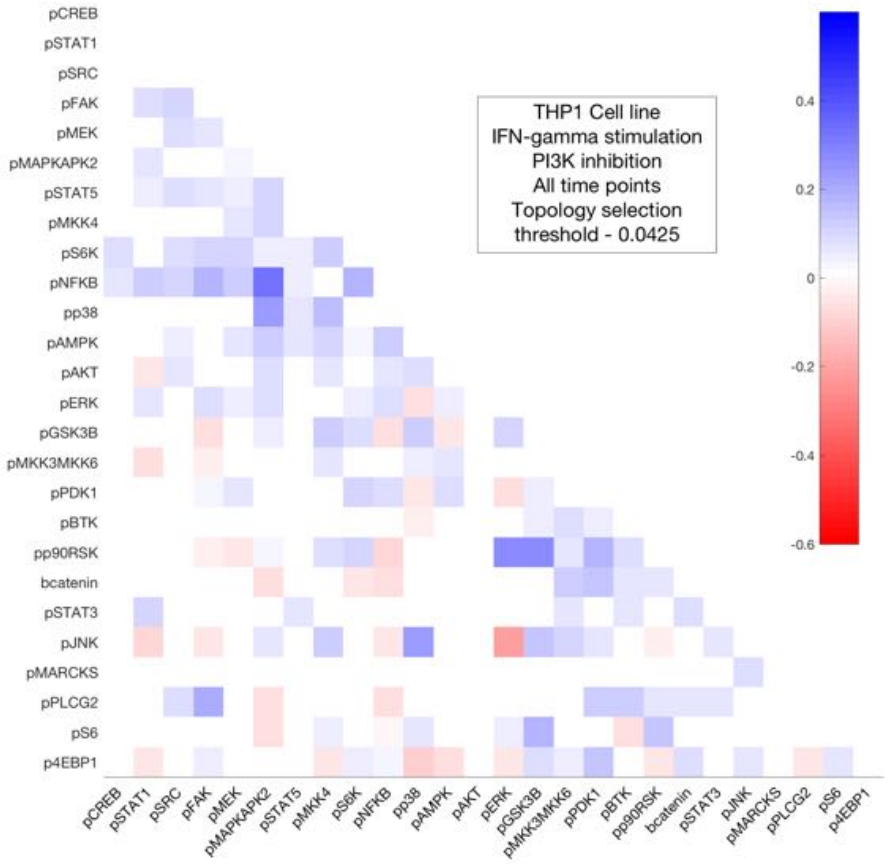

Figure S.11.8: Estimated partial correlation matrix from PI3K inhibited experiment.

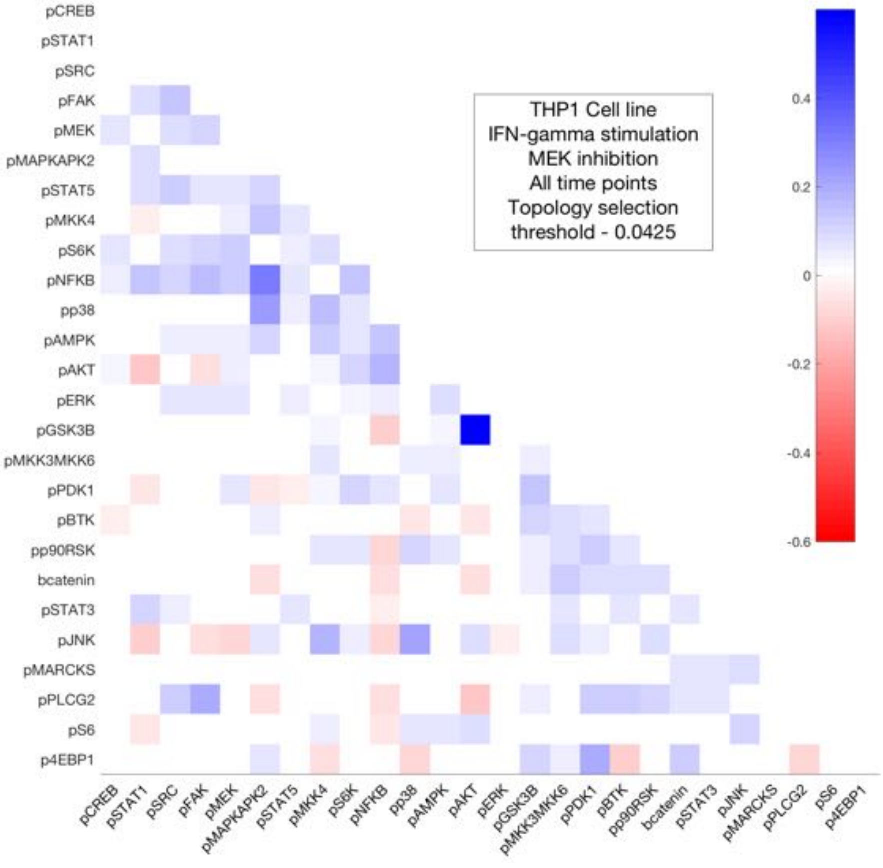

Figure S.11.9: Estimated partial correlation matrix from MEK inhibited experiment.

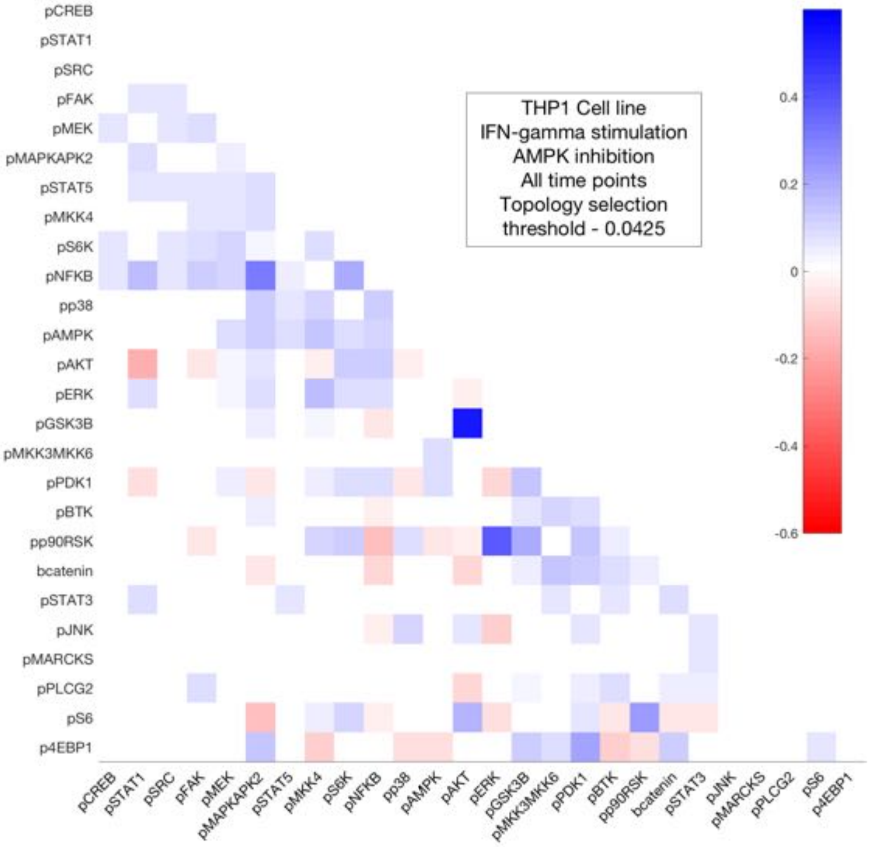

Figure S.11.10: Estimated partial correlation matrix from AMPK inhibited experiment.

SI.11.3 Literature based validation

Table S.11.1: Literature based validation. The table shows the set of predicted edges that are validated through Omnipath database.

**No.**	**Undirected Relationship**	**Validated**	**Shortest Length**	**Intermediate Protein**	**PubMed ID**
1	pP90RSK - pS6	MEK	1		17360704, 21233202
2	pAKT - pPLCγ2	PI3K	2	SRC, EGFR	18283331, 11606584
3	pAKT - pS6	PI3K	2	S6K	15149849, 17360704
4	pAKT - pGSK3β	PI3K	1		11035810, 8524413
5	pERK - pP90RSK	MEK	1		12832467
6	pERK - pJNK	MEK	1		15778365
7	pFAK - pNFκB	AMPK	2	STAT1/3, TP53, GSK3B	11278462, 16481475
8	pFAK - pPLCγ2	AMPK	2	SRC, EGFR, GRB2	17828307, 15735019, 11606584
9	pGSK3β - pPDPK1	PI3K	1		9373175
10	pGSK3β - pP90RSK	MEK	1		1137602
11	pMAPKAPK2 - pMKK4		2	p38, AKT	14499342, 7535770
12	pMAPKAPK2 - pP38	AMPK	1		14499342
13	pMAPKAPK2 - pNFκB		2	ERK, p38	8846784, 15073167
14	pMKK3&6 - β-CATENIN		2	JNK, NFkB, SMAD7	16498455, 22328140
15	pMKK4 - pJNK	AMPK	1		12788955, 10715136, 8974401
16	pMKK4 - pAMPK		2	MAP3K7	9278437, 20615388
17	pMKK4 - pP38		1		12788955
18	pNFκB - pP90RSK		2	ERK, GSK3B	17183360, 11584304
19	pP38 - pJNK	AMPK	1		15778365
20	pPDPK1 - pP90RSK		1		10480933

No.	Undirected Relationship	Validated	Shortest Length	Intermediate Protein	PubMed ID
21	pPDPK1 - p4EBP1		2	AKT1, S6K	12167717, 11777913
22	pS6K - pNFκB		2	MDM2, MAPK1	7545671, 21035469, 15073167
23	pSRC - pFAK	AMPK	1		17828307, 15735019
24	pSTAT1 - pAKT	PI3K	1		15284024
25	pSTAT1 - pP38		1		17502367
26	pSTAT1 - pERK	MEK	1		7569900
27	pSTAT1 - pNFκB		1		16481475
